# The ALS- and FTD-associated proteins annexin A11 and CHMP2B act sequentially in plasma membrane repair

**DOI:** 10.1016/j.devcel.2026.05.014

**Published:** 2026-07-08

**Authors:** Catherine M. Heffner, Georgina P. Starling, Lorian C. Straker, Philippa C. Hawes, Adrian M. Isaacs, Jeremy G. Carlton

**Affiliations:** 1 Organelle Dynamics Laboratory, The Francis Crick Institute, 1 Midland Road, London NW1 1AT, UK; 2 School of Cancer & Pharmaceutical Sciences, King’s College London, London SE1 1UL, UK; 3 UK Dementia Research Institute at UCL, London WC1E 6BT, UK; 4 Department of Neurodegenerative Disease, UCL Queen Square Institute of Neurology, London WC1N 3BG, UK; 5 Electron Microscopy Science Technology Platform, The Francis Crick Institute, 1 Midland Road, London NW1 1AT, UK

**Keywords:** ESCRT-III, CHMP2B, ANXA11, membrane repair, annexin, FTD, ALS, pore-forming toxin

## Abstract

Maintenance of plasma membrane integrity is essential for compartmentalization of the cytosol and for cellular viability. Upon membrane damage, several factors including endosomal sorting complex required for transport-III (ESCRT-III) proteins, annexins, stress granules, lipids, and membrane fusion proteins are mobilized to orchestrate membrane repair. However, whether these factors operate independently or act together is unclear. Here, using human cell lines, we expose temporal differences and interdependencies in the recruitment of ESCRT-III and annexin proteins to sites of plasma membrane damage. We show that annexin proteins are recruited immediately and form a plug at the damage site, restricting membrane permeability. We find that ESCRT-III assembles later and acts to release plug-containing damaged membranes from the cell. Further, frontotemporal dementia (FTD)- and amyotrophic lateral sclerosis (ALS)-associated mutations in the ESCRT-III protein, CHMP2B, and the annexin protein, ANXA11, compromise plasma membrane repair, suggesting that defects in this process may contribute to these pathologies. These data present an integrated “sealing and healing” model of membrane repair.

## Highlights

• ANXA11 rapidly localizes to and plugs holes in damaged plasma membranes
• CHMP2B localizes later to sites of damage and helps externalize ANXA11 plugs
• Shedding of these lesions heals the membrane and preserves cellular viability
• Some FTD/ALS-associated mutations in ANXA11 and CHMP2B impair membrane repair

## Graphical abstract

**Figure:**
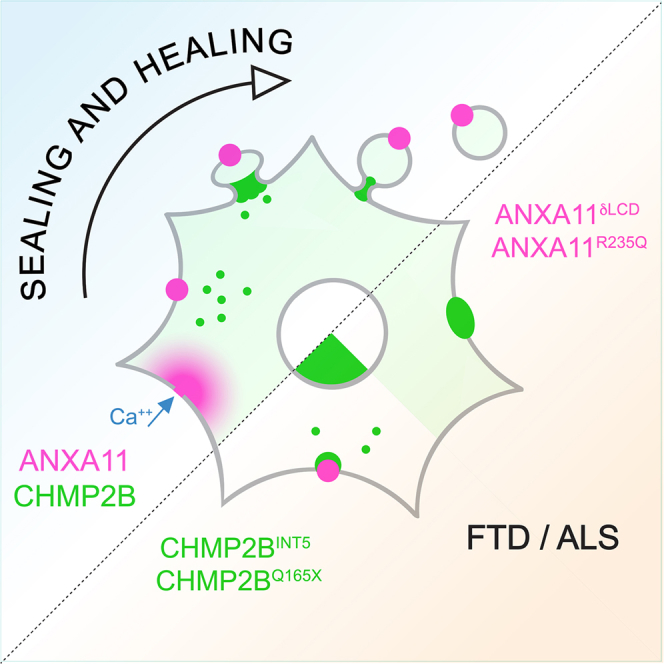

## Teaser

Heffner et al. show that ANXA11 and CHMP2B, proteins encoded by ALS- and FTD-risk genes, are sequentially recruited to sites of plasma membrane damage. Membrane repair involves ANXA11-positive plug formation and ESCRT-III-dependent extrusion via an integrated ‘sealing and healing’ mechanism. Disease-associated ANXA11 and CHMP2B mutations similarly stall ESCRT-III during membrane repair.

## Introduction

The maintenance of plasma membrane integrity is essential for cellular viability and cytosolic compartmentalization. This integrity can be compromised by a range of insults, including physical forces, crystalline material, protein aggregates, reactive oxygen species, and bacterially secreted pore-forming toxins (PFTs).^1^^,^^2^ Indeed, several cellular components of immune control such as perforins,^3^ gasdermins,^4^^,^^5^^,^^6^^,^^7^^,^^8^ ninjurin-1,^9^^,^^10^ and the complement membrane attack complex^11^ exploit this vulnerability to purposefully damage the plasma membrane and compromise viability. To counter these insults, cells must sense membrane damage and mobilize factors to orchestrate a lasting repair. Several molecular systems have been proposed to contribute to membrane repair, and current models include endosomal sorting complex required for transport-III (ESCRT-III)-dependent sealing,^12^^,^^13^ annexin- and apoptosis linked gene-2 (ALG-2)-dependent sealing,^14^^,^^15^^,^^16^ the plugging of membrane lesions with stress granules,^17^ repair of damage through organelle fusion and lipid remodeling,^18^ the internalization and degradation of damaged membranes,^19^^,^^20^^,^^21^ and the release of damaged membranes through microvesicles.^15^^,^^16^^,^^22^ Whether these activities represent independent membrane repair mechanisms or are integrated parts of a unified repair process is unclear.

ESCRT-III is an ancient membrane-remodeling complex with essential roles in several membrane-shaping processes, including endosomal sorting, exosome biogenesis, enveloped virus release, cytokinetic abscission, mitotic nuclear envelope regeneration, and the repair of ruptured nuclear, plasma and lysosomal membranes.^23^^,^^24^^,^^25^^,^^26^ Mammalian cells express 11 ESCRT-III subunits called charged multivesicular body proteins (CHMPs) and a related protein called increased sodium tolerance-1 (IST-1). Alongside the AAA-ATPase, vacuolar protein sorting-associated protein 4 (VPS4), whose microtubule interaction and trafficking (MIT)-domain binds MIT-domain interaction motifs (MIMs) in the C terminus of ESCRT-III proteins, ESCRT-III remodels and separates cellular membranes that bud away from the cytosol.^27^^,^^28^ C-terminal truncating mutations in *CHMP2B* that arise through mutation of a splice acceptor site and retention of intron 5 (CHMP2B^INT5^), or from a *de novo* termination codon in exon 4 (CHMP2B^Q165X^), cause a rare form of autosomal dominant frontotemporal dementia (FTD).^29^^,^^30^^,^^31^ Several other ESCRT-associated proteins have been implicated in a range of neurodegenerative diseases and neurodevelopmental disorders, suggesting a wider implication of ESCRT activity in neurological health.^32^^,^^33^^,^^34^^,^^35^^,^^36^ The calcium-dependent phospholipid binding annexin proteins have also been implicated in membrane repair at plasma, endosomal, and nuclear membranes.^15^^,^^16^^,^^37^^,^^38^^,^^39^^,^^40^ Mutations in *Annexin A11* (*ANXA11*) are associated with amyotrophic lateral sclerosis (ALS),^41^ a disease with genetic, clinical, and neuropathological overlap with FTD.^42^ Given the identification of membrane-damaging properties in several protein aggregates associated with neurodegenerative disease, such as amyloid-β,^43^^,^^44^^,^^45^ α-synuclein,^43^^,^^46^^,^^47^ and tau-fibrils,^48^ and recent suggestions that membrane damage is at the core of Alzheimer’s disease,^49^ we wondered if these different FTD/ALS risk factors operated at membranes in a common pathway to preserve cellular viability.

Here, we demonstrate sequential recruitment of ANXA11 and CHMP2B to sites of plasma membrane damage, with ANXA11 arrival coincident with the formation of a plug in the damaged membrane that restricts membrane permeability, while CHMP2B arrives after membrane sealing has occurred to allow extrusion and release of membranes containing this plug. Further, we show that FTD-causing mutations in *CHMP2B* and some pathogenic mutations in *ANXA11* cause a phenotypically similar stalling of ESCRT-III turnover at sites of membrane damage. These data provide an integrated “sealing and healing” model for the repair of damaged cellular membranes and describe a common mechanistic phenotype associated with separate FTD/ALS-associated mutations.

## Results

### FTD-associated mutations in CHMP2B cause its nuclear retention

**Figure 1:**
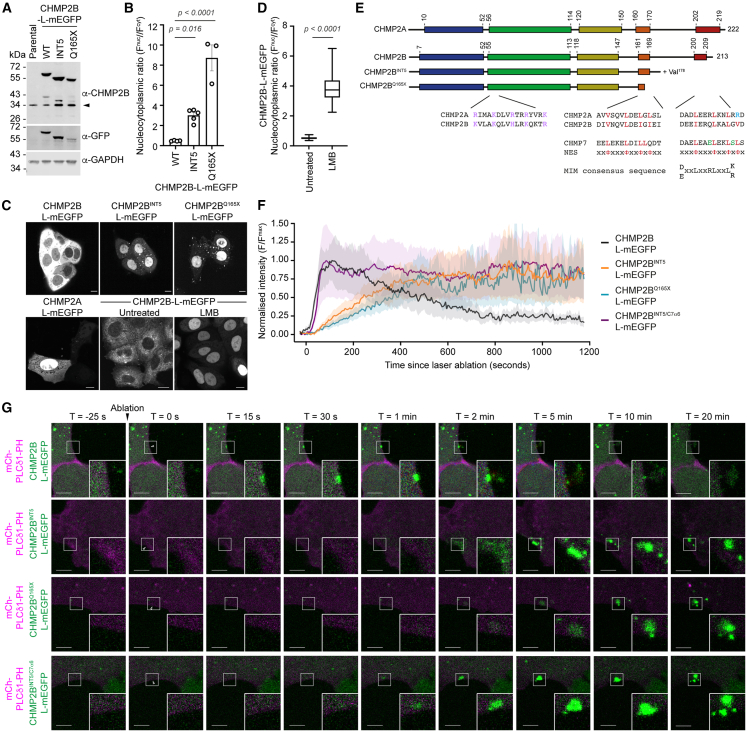
Pathogenic variants of CHMP2B truncate a C-terminal NES and compromise recruitment to damaged plasma membrane (A) Resolved cell lysates of CAL-51 cells stably expressing CHMP2B-L-mEGFP, CHMP2B^INT5^-L-mEGFP, or CHMP2B^Q165X^-L-mEGFP were examined by western blotting with antibodies that recognise CHMP2B, GFP, or GAPDH. Arrowhead indicates endogenous CHMP2B. (B) Quantification of nucleocytoplasmic fluorescence from cells stably expressing CHMP2B-L-mEGFP (*n* = 57, *N* = 4), CHMP2B^INT5^-L-mEGFP (*n* = 31, *N* = 5), CHMP2B^Q165X^-L-mEGFP (*n* = 104, *N* = 3). Mean ± SEM with significance calculated by one-way ANOVA. (C) Live imaging of CAL-51 cells stably expressing CHMP2B-L-mEGFP, CHMP2B^INT5^-L-mEGFP, CHMP2B^Q165X^-L-mEGFP, or CHMP2A-L-mEGFP and treated with LMB (10 ng/mL, 4 h) as indicated. Scale bar is 10 μm. (D) Quantification of the nucleocytoplasmic ratio of CHMP2B-L-mEGFP (*n* = 47 [untreated] or 71 [+ LMB] cells). Data displayed as a box and whisker plot with median, 25^th^ and 75^th^ percentile displayed. Significance calculated by two-tailed *t* test. (E) Schematic representation of CHMP2A, CHMP2B, CHMP2B^INT5^, and CHMP2B^Q165X^. Colored blocks represent helix boundaries obtained from Alphafold2 predictions of CHMP2A (O43633) or CHMP2B (Q9UQN3). Basic residues forming the putative nuclear retention sequence in Helix2 of CHMP2 proteins highlighted in lilac. Hydrophobic residues forming nuclear export sequences (NES) in CHMP2B’s C-terminal helices highlighted in red, alongside equivalent residues from the CHMP7 NESs. The hydrophobic-to-basic amino acid change that inactivates CHMP2A’s Helix6 NES highlighted in blue. MIT-domain interaction motif (MIM) consensus sequence and residues in Helix6 of CHMP2A and CHMP2B, with amino acids that inactivate CHMP7’s MIM^51^ highlighted in green. (F and G) Quantification (F) and representative image sequences (G) of CAL-51 cells stably expressing CHMP2B-L-mEGFP, CHMP2B^INT5^-L-mEGFP, CHMP2B^Q165X^-L-mEGFP, or CHMP2B^INT5/C7α6^-L-mEGFP and subject to laser ablation at the mCh-PLCδ1-PH-decorated plasma membrane. Arrowheads indicate site of laser ablation at T = 0. Separated channels are displayed in Figure S3E. Scale bar is 5 μm. Graph displays mean ± SEM from CHMP2B-L-mEGFP (*n* = 14, *N* = 7), CHMP2B^INT5^-L-mEGFP (*n* = 11, *N* = 5), CHMP2B^Q165X^-L-mEGFP (*n* = 12, *N* = 6), or CHMP2B-L-mEGFP, CHMP2B^INT5/C7α6^-L-mEGFP (*n* = 12, *N* = 6). See also Figures S1–S3 and Video S1.

We generated CAL-51 cells stably expressing low levels of human CHMP2 proteins tagged at their Cterminus with monomeric enhanced green fluorescent protein (mEGFP) via a flexible linker (L) from the localization and purification (LAP) tag^50^ (Figure 1A). As expected, while CHMP2B-L-mEGFP was cytoplasmic, CHMP2B^INT5^-L-mEGFP and CHMP2B^Q165X^-L-mEGFP both localized to aberrant endosomes.^31^^,^^52^^,^^53^ However, we noticed that CHMP2B^INT5^-L-mEGFP and CHMP2B^Q165X^-L-mEGFP were mislocalized to the nucleus (Figures 1B and 1C). We confirmed these localisations by analyzing hemagglutinin (HA)-tagged proteins (Figures S1A and S1B) and CAL-51 cells edited to express CHMP2B-L-mClover3^+/+^ or CHMP2B^INT5^-L-mClover3^+/+^ (Figure S1C–S1E). Extending these observations, we found that the CHMP2B paralog, CHMP2A-L-mEGFP, localized strongly to the nucleus (Figure 1C). These data show that the steady-state nucleocytoplasmic distribution of CHMP2A and CHMP2B proteins is non-equivalent and that FTD-causing mutations in CHMP2B lead to its nuclear retention.

Partitioning between nucleus and cytoplasm is governed by the nucleocytoplasmic transport machinery. Inhibition of the exportin CRM1/XPO1 with leptomycin B (LMB) resulted in nuclear accumulation of CHMP2B-L-mEGFP (Figures 1C and 1D) but had limited effect on the nuclear localization of CHMP2B^INT5^-L-mEGFP and CHMP2B^Q165X^-L-mEGFP (Figures S1F–S1H). As such, we conclude that C-truncating mutations in CHMP2B restrict its nuclear export. Type-1 nuclear export sequences (NESs) have been identified in Helix5 and Helix6 of CHMP7, which limit its exposure to inner nuclear membrane proteins such as LEMD2.^51^^,^^54^^,^^55^ We noted similar sequences in the C terminus of CHMP2B that were truncated in CHMP2B^INT5^ and CHMP2B^Q165X^ (Figure 1E). By individually inactivating these sequences, we found that both acted as NESs, with the C-terminal NES contributing more transport activity than the internal NES (Figures S1I and S1J). CHMP2A encodes a basic residue instead of a hydrophobic residue at position 221 in this C-terminal NES (Figure 1E), and we hypothesized that this would inactivate its transport activity. We mutated the equivalent residue in CHMP2B (Val212) and found that CHMP2B^V212K^-L-mEGFP was poorly exported from the nucleus (Figures S1I and S1J). We next fused the Helix6 NES from CHMP7 to the C terminus of CHMP2B^INT5^-L-mEGFP or CHMP2B^Q165X^-L-mEGFP (Figure S1K) and recovered CHMP2B’s cytoplasmic localization (Figures S1I and S1J). Finally, fusing the CHMP7 NES to CHMP2A’s C terminus (CHMP2A^C7α6^-L-mEGFP) or mutating the basic residue at position 221 to a hydrophobic residue (CHMP2A^R221L^-L-mEGFP) enabled nuclear export of CHMP2A (Figures S1L and S1M), indicating that its nuclear accumulation was due to the absence of NES activity.

The LMB-sensitive nuclear export of CHMP2B-L-mEGFP and the nuclear accumulation of CHMP2A-L-mEGFP, CHMP2B^INT5^-L-mEGFP, CHMP2B^Q165X^-L-mEGFP, and CHMP2B^V212K^-L-mEGFP suggested the presence of nuclear localization determinants within these proteins. Using an existing deletion series of YFP-CHMP2A,^56^ we identified that residues 64–74 in CHMP2A’s N terminus were needed for nuclear localization (Figures S2A–S2D). By inspecting Alphafold-2^57^ models of CHMP2A and CHMP2B, we noticed a cluster of surface-exposed basic residues in this region (Figure S2E). Inactivating these residues prevented nuclear accumulation of CHMP2A-L-mEGFP, CHMP2B-L-mEGFP, or CHMP2B^INT5^-L-mEGFP (Figures S2F and S2G). As a bipartite nuclear localization sequence (NLS) in CHMP1 proteins has been described,^58^ and other ESCRT-III subunits such as CHMP4B display nuclear enrichment,^59^ we wondered if these sequences encoded a NLS. While expression of RAN^Q69L^-mRFP or RAN^T24N^-mRFP prevented nuclear localization of GFP-NLS^SV40^-β-galactosidase, it had little effect on the localization of CHMP2 proteins (Figures S2H and S2I), and unlike canonical NLSs, the isolated CHMP2 sequences provided no transport activity when fused to GFP-β-galactosidase (Figures S2J and S2K). In summary, these data identify N-terminal nuclear retention sequences in CHMP2 proteins that are balanced by a powerful C-terminal NES in CHMP2B but not in CHMP2A (Figure 1E), explaining the differences in nucleocytoplasmic distribution between these proteins.

### Assembly of FTD-associated mutants of CHMP2B at sites of plasma membrane damage is delayed and persistent

We next asked whether the nuclear retention of C-truncated mutants of CHMP2B affected its ability to respond to plasma membrane damage. We imaged cells stably expressing CHMP2B-L-mEGFP and used a multi-photon laser to ablate a single pixel at the plasma membrane. Confirming known roles of ESCRT-III in plasma membrane repair,^12^^,^^13^ we observed transient assembly and disassembly of CHMP2B-L-mEGFP at the site of damage (Figures 1F and 1G; Video S1). Interestingly, when the site of ablation was placed at the edge of a lamellipodia, we visualized a “wave” of CHMP2B-L-mEGFP that traveled progressively toward the site of ablation and narrowed to a focus at the ablation site (Figure S3A; Mendeley Data Video 1). While prior data have implicated actin remodeling^22^ and lysosomal fusion^18^ in plasma membrane repair, we saw no evidence of actin or lysosome relocalisation, and CHMP2B-L-mEGFP recruitment was insensitive to actin cytoskeleton disruption (Figures S3B–S3D, Mendeley Data Videos 2–4). We next examined recruitment of CHMP2B^INT5^-L-mEGFP and CHMP2B^Q165X^-L-mEGFP to sites of plasma membrane damage. Consistent with a limited cytosolic pool of these proteins, both accumulated more slowly at sites of damage (Figures 1F, 1G, and S3E; Video S1). Cells stably expressing a chimaera of CHMP2B^INT5^ and the Helix6 NES from CHMP7 (CHMP2B^INT5/C7α6^-L-mEGFP; Figure S1K) showed that returning this protein to the cytosol restored its timely assembly at sites of plasma membrane damage (Figures 1F, 1G, and S3E; Video S1). While recruitment of CHMP2B-L-mEGFP to sites of plasma membrane damage was transient, recruitment of CHMP2B^INT5^-L-mEGFP and CHMP2B^Q165X^-L-mEGFP was persistent (Figures 1F, 1G, and S3E). As these C-truncating mutations remove the VPS4-binding MIM,^52^^,^^53^ we wondered whether a failure to bind VPS4 was responsible for the impaired turnover of these mutants. Charge inversions in CHMP7’s Helix6 have inactivated its MIM,^51^ and consistent with a role for VPS4 in ESCRT-III turnover, while CHMP2B^INT5/C7α6^-L-mEGFP was recruited on schedule, it was not disassembled after recruitment (Figures 1F, 1G, and S3E; Video S1, Mendeley Data Video 5). Reverting these charges to restore VPS4 binding^51^ recovered the turnover of CHMP2B^INT5/C7α6(ES-RR)^-L-mEGFP at sites of plasma membrane damage (Figures S3F and S3G; Mendeley Data Video 6). These data separate NES and MIM activities in CHMP2B’s C terminus, with the NES allowing cytoplasmic access for timely recruitment to sites of action and the MIM allowing VPS4-binding for turnover once assembled.

Video S1. Recruitment of CHMP2B-L-mEGFP, CHMP2B^INT5^-L-mEGFP, CHMP2B^Q165X^-L-mEGFP, and CHMP2B^INT5/C7α6^-L-mEGFP to sites of plasma membrane damage, related to Figure 1 CAL-51 cells stably expressing the indicated CHMP2B-L-mEGFP proteins and transfected with mCh-PLCδ1-PH were imaged live and subject to a single pixel multi-photon laser ablation at the plasma membrane (T = 0 seconds, site marked by arrowhead). Frames were acquired every 5 seconds. Videos are concatenated. CHMP2B-L-mEGFP (n = 14, N = 7), CHMP2B^INT5^-L-mEGFP (n = 11, N = 5), CHMP2B^Q165X^-L-mEGFP (n = 12, N = 6) and CHMP2B^INT5/C7α6^-L-mEGFP (n = 12, N = 6). In videos, scale bar is 5 μm. See also Figure S3E for separated channels.

### ESCRT-III recruitment is temporally disconnected from membrane sealing

**Figure 2:**
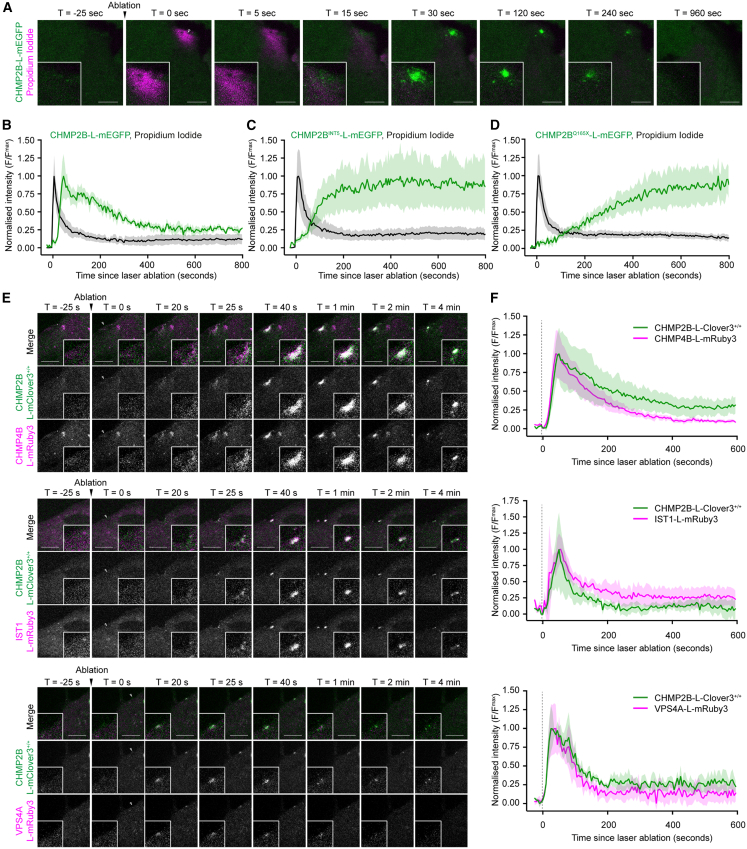
ESCRT-III recruitment occurs after membrane impermeability has been restored (A) Representative image sequences of CAL-51 cells stably expressing CHMP2B-L-mEGFP and subject to laser ablation in the presence of external propidium iodide (PI, 160 μg/mL, magenta). See corresponding Video S2. Scale bar is 5 μm. (B–D) Quantification of PI influx (black) and recruitment dynamics of CHMP2B-L-mEGFP (*n* = 15, *N* = 5), CHMP2B^INT5^-L-mEGFP (*n* = 8, *N* = 4), and CHMP2B^Q165X^-L-mEGFP (*n* = 12, *N* = 7). Graphs display mean ± SEM. (E and F) Representative image sequences (E) and quantification (F) of CAL-51 cells edited to express CHMP2B-L-mClover3^+/+^ and stably transduced with CHMP4B-L-mRuby3 (*n* = 9, *N* = 3), IST1-L-mRuby3 (*n* = 9, *N* = 3), or VPS4A-L-mRuby3 (*n* = 8, *N* = 3) and subject to laser ablation at the plasma membrane. See corresponding Mendeley Data Videos 7–9. Graphs display mean ± SEM. In images, arrowhead indicates site of laser ablation at T = 0. Scale bar is 10 μm.

Given the delay in recruitment of C-truncated versions of CHMP2B to sites of membrane damage, we wondered when membrane integrity was restored relative to the ablation. Propidium iodide (PI) is a nucleic acid binding dye that is frequently used as a whole-cell reporter of plasma membrane damage.^60^^,^^61^ Ablation of a single pixel at the plasma membrane resulted in a local burst of PI influx (Figures 2A and 2B; Video S2). As PI entry was transient, rather than progressive, we infer that permeability to small molecules was rapidly restricted after ablation. While previous studies suggested that ESCRT-III recruitment occurred concomitantly with plasma membrane sealing,^12^ our imaging revealed that CHMP2B-L-mEGFP was recruited to sites of plasma membrane damage only after PI influx had abated (Figures 2A and 2B; Video S2). Consistent with ESCRT-III being dispensable for the restriction of membrane permeability, the kinetics of PI influx after damage was unchanged in cells expressing CHMP2B^INT5^-L-mEGFP or CHMP2B^Q165X^-L-mEGFP, despite these mutants being delayed in their recruitment to sites of plasma membrane damage (Figures 2C and 2D). We investigated recruitment kinetics of endogenous CHMP2B-L-mClover3^+/+^ at sites of laser ablation in context with other ESCRT-III proteins and confirmed co-recruitment with CHMP4B-L-mRuby3, IST1-L-mRuby3, and VPS4A-L-mRuby3 (Figures 2E and 2F; Mendeley Data Videos 7–9), indicating that the kinetics of CHMP2B recruitment reflect that of the broader ESCRT-III pathway. These data indicate that the restriction of membrane permeability and the recruitment of ESCRT-III are temporally disconnected and suggest that ESCRT-III-independent factors are responsible for the acute seal of damaged membranes.

Video S2. PI entry after laser ablation is transient and abates before CHMP2B-L-mEGFP is recruited to sites of plasma membrane damage, related to Figure 2 CAL-51 cells stably expressing CHMP2B-L-mEGFP were imaged live in the presence of 160 μg/ml PI and subject to a single pixel multi-photon laser ablation at the plasma membrane (T = 0 seconds, site marked by arrowhead). Frames were acquired every 5 seconds, n = 15 videos, N = 5. Scale bar is 5 μm.

### ANXA7 and ANXA11 are recruited immediately to seal damaged plasma membranes

**Figure 3:**
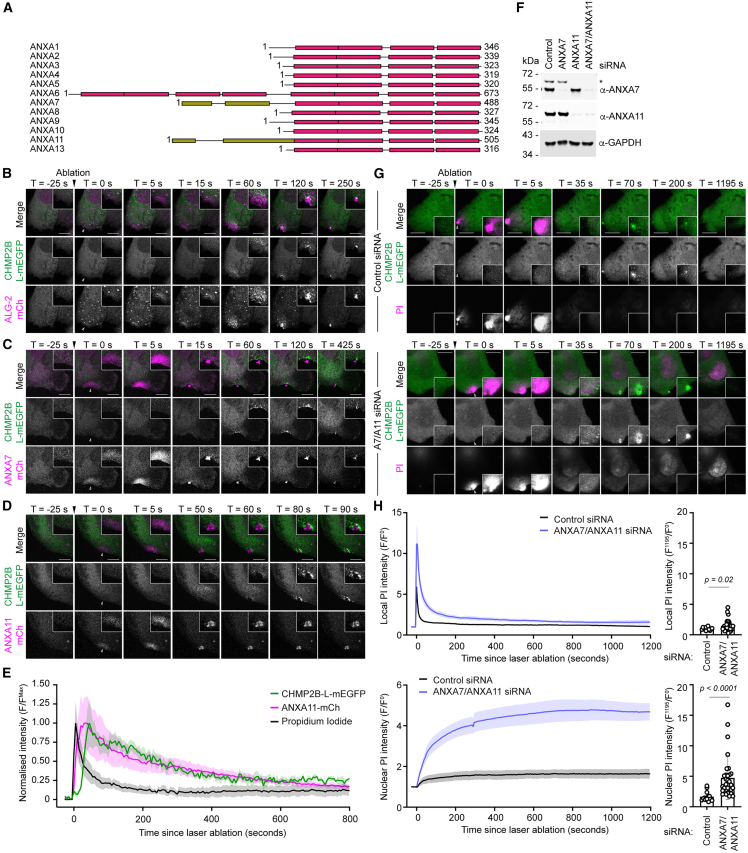
ALG-2, ANXA7, and ANXA11 are recruited to sites of membrane damage before CHMP2B (A) Schematic of sequences of human annexin proteins A1–A13. Annexin-like domain shown in pink, LCD shown in gold. (B–D) Representative image sequences of CAL-51 cells stably expressing CHMP2B-L-mEGFP and either ALG-2-mCh (*n* = 5, *N* = 2), ANXA7-mCh (*n* = 8, *N* = 3), or ANXA11-mCh (*n* = 11, *N* = 3) and subject to laser ablation at the plasma membrane. Arrowheads indicate site of laser ablation at T = 0. Scale bar is 10 μm. See corresponding Video S3. (E) Overlay of PI-influx dynamics and CHMP2B-L-mEGFP and ANXA11-mCh recruitment dynamics downstream of laser ablation. The average ANXA11-mCh (*n* = 6, *N* = 3) trace was overlaid on the CHMP2B-L-mEGFP and PI traces (*n* = 15, *N* = 5) from Figure 2B. Data displayed as mean ± SEM. (F) Resolved cell lysates of CAL-51 cells transfected with the indicated siRNA were examined by western blotting with antibodies that recognise ANXA7, ANXA11 or GAPDH. Asterisk indicates detection of an ANXA11-siRNA sensitive band with anti-ANXA7 antibodies. (G) Representative image sequences of CAL-51 cells stably expressing CHMP2B-L-mEGFP and transfected with the indicated siRNA and subject to laser ablation at the plasma membrane in the presence of external PI (160 μg/mL). Scale bar is 10 μm. (H) Quantification of PI entry at the site of damage or in the nucleus from cells in (G). Traces show mean ± SEM from control (*n* = 14, *N* = 5) or ANXA7/ANXA11-depleted (*n* = 31, *N* = 5) conditions. Bar charts show mean ± SD of the PI intensity the site of damage or in the nucleus from individual cells in (F) after 20 min. Significance calculated by two-tailed *t* test across individual data points. See also Figure S4 and Video S4.

We looked upstream at potential recruitment factors for ESCRT-III. Human cells express 12 annexin proteins (Figure 3A) that bind membranes in a calcium-dependent manner. ANXA7 and ANXA11 share an extended N-terminal low-complexity domain (LCD) that binds the ESCRT-III-associated factor ALG-2^62^^,^^63^ and that participates in biomolecular condensate formation.^64^^,^^65^ We generated cells stably expressing both mCherry (mCh)-tagged versions of these proteins and CHMP2B-L-mEGFP. Building on the discovery that ALG-2 and ANXA7 were recruited rapidly to sites of plasma membrane damage prior to ESCRT-III,^14^ we confirmed that ALG-2 was enriched progressively at the point of damage, peaking just before CHMP2B-L-mEGFP and forming a cluster that protruded from the cell (Figure 3B; Video S3). Like ANXA7-mCh (Figure 3C; Video S3), ANXA11-mCh was recruited immediately to sites of damage, first decorating a broad area of the plasma membrane and rapidly becoming focused to a point at the site of ablation that also protruded from the cell (Figure 3D; Video S3). CHMP2B-L-mEGFP was recruited some 40 s later, initially covering a broad area of the plasma membrane surrounding the annexin (Figures 3C and 3D) before being brought to a focus at the ablation site. Importantly, ANXA7-mCh and ANXA11-mCh recruitment was temporally co-ordinated with the block in PI influx (Figure 3E), and depletion of ANXA7 and ANXA11 led to both a greater initial PI influx and persistent PI entry, suggesting that these annexins contribute to the acute restriction of permeability at damaged plasma membranes (Figures 3F–3H; Video S4).

Video S3. Temporal differences between recruitment of ALG-2, ANXA7, ANXA11, and CHMP2B, related to Figure 3 CAL-51 cells stably expressing CHMP2B-L-mEGFP and the indicated mCherry fusion proteins were imaged live and subject to a single pixel multi-photon laser ablation at the plasma membrane (T = 0 seconds, site marked by arrowhead). Frames were acquired every 5 seconds, CHMP2B-L-mEGFP and ALG-2-mCh (n = 5 videos, N = 2), CHMP2B-L-mEGFP and ANXA7-mCh (n = 8 videos, N = 3), CHMP2B-L-mEGFP and ANXA11-mCh (n = 11 videos, N = 3). In videos, scale bar is 5 μm.

Video S4. Loss of ANXA7 and ANXA11 impairs plasma membrane sealing after damage, related to Figure 3 CAL-51 cells stably expressing CHMP2B-L-mEGFP were treated with control siRNA or ANXA7- and ANXA11-targeting siRNA, imaged live and subject to a single pixel multi-photon laser ablation at the plasma membrane (T = 0 seconds, site marked by arrowhead) in the presence of PI (160 μg/mL). Frames were acquired every 5 seconds. Control siRNA (n = 14 videos, N = 5), ANXA7/ANXA11 siRNA (n = 31 videos, N = 5). In videos, scale bar is 5 μm.

### Annexin recruitment is needed for ESCRT-III turnover at sites of plasma membrane damage

**Figure 4:**
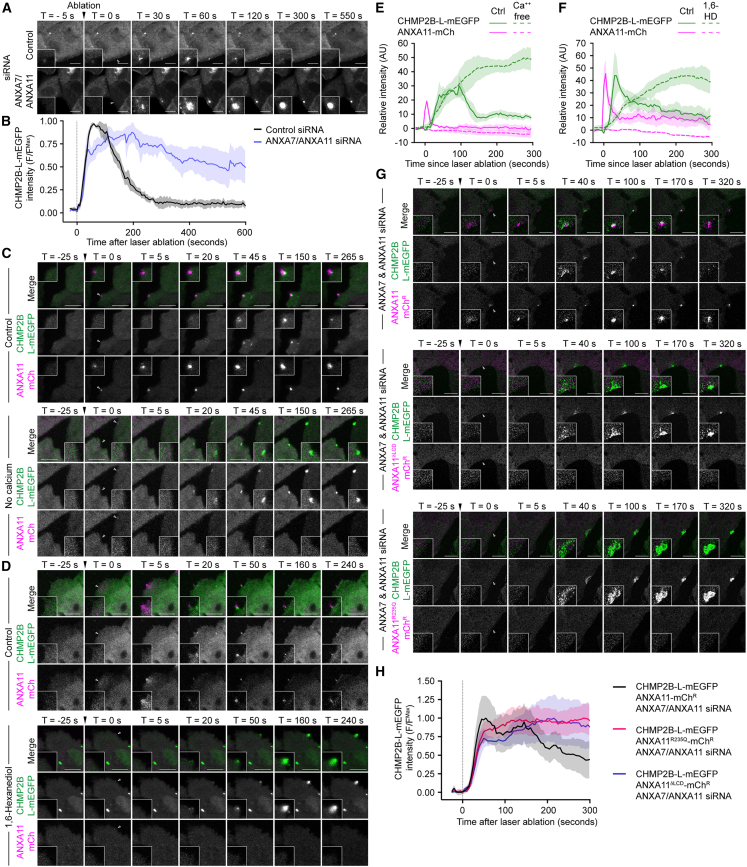
Impairing ANXA11-assembly at sites of plasma membrane damage prevents CHMP2B-L-mEGFP turnover (A) Representative image sequences of CAL-51 cells stably expressing CHMP2B-L-mEGFP, transfected with the indicated siRNA and subject to laser ablation at the plasma membrane. Scale bar is 10 μm. (B) Quantification of CHMP2B-recruitment in cells from (A) (control siRNA, *n* = 10, *N* = 3; ANXA7/ANXA11 siRNA, *n* = 8, *N* = 3), see also Mendeley Data Videos 10 and 11. Graph displays mean ± S.E.M. (C and D) Representative images of CAL-51 cells stably expressing CHMP2B-L-mEGFP and ANXA11-mCh and imaged in the absence of calcium (C), or in the presence of 1.25% 1,6-hexanediol (D). Scale bar is 10 μm. (E and F) Quantification of CHMP2B-L-mEGFP and ANXA11-mCh recruitment in cells from (C) and (D). Graphs display mean ± SEM. In (E) (control, *n* = 11, *N* = 3; calcium free, *n* = 17, *N* = 3). In (F) (control, *n* = 11, *N* = 3; 1,6-hexanediol, *n* = 21, *N* = 3), see also Mendeley Data Videos 12–14. (G) Representative image sequences from CAL-51 cells treated with siRNA-targeting ANXA7 and ANXA11, stably expressing CHMP2B-L-mEGFP and either siRNA resistant ANXA11-mCh, ANXA11^R235Q^-mCh, or ANXA11^δLCD^-mCh and subject to a single multi-photon laser ablation at the plasma membrane, indicated by the arrowhead. Scale bar is 10 μm. (H) Quantification of recruitment and turnover of CHMP2B-L-mEGFP in the cell lines described in (G). Mean ± SEM displayed from ANXA7/ANXA11 siRNA treated CHMP2B-L-mEGFP:ANXA11-mCh cells (*n* = 10, *N* = 4); CHMP2B-L-mEGFP:ANXA11^R235Q^-mCh cells (*n* = 11, *N* = 4); and CHMP2B-L-mEGFP:ANXA11^δLCD^-mCh cells (*n* = 14, *N* = 4). See corresponding Video S5 and related data in Figure S5.

We next examined ESCRT-III recruitment in the absence of ANXA7 and ANXA11. We were surprised to find that CHMP2B-L-mEGFP was recruited on schedule but was not disassembled in ANXA7/ANXA11-depleted cells (Figures 4A and 4B; Mendeley Data Videos 10 and 11). We looked to test which activities were needed for annexin and ESCRT recruitment. As expected, in cells stably expressing ANXA11-mCh and CHMP2B-L-mEGFP, external calcium was necessary for ANXA11-mCh recruitment to sites of plasma membrane damage (Figures 4C and 4E). Surprisingly, calcium removal had no effect on the recruitment of CHMP2B-L-mEGFP, but prevented its turnover. Similarly, in the presence of 1,6-hexanediol, a small molecule that disrupts formation of biomolecular condensates, ANXA11-mCh did not assemble at sites of plasma membrane damage (Figures 4D and 4F), and CHMP2B-L-mEGFP was again recruited but not turned over (Figures 4C–4F; Mendeley Data Videos 12–14). In the absence of external calcium, there was a catastrophic failure to restrict PI entry after ablation, whereas in 1,6-hexanediol-treated cells, the failure to restrict PI entry after ablation was more modest, mimicking the effects of ANXA7 and ANXA11 depletion (Figures S4A and S4B; Mendeley Data Videos 15–17). These data expose separate calcium-dependent and calcium-independent recruitment cues for ANXA11 and CHMP2B respectively, showing that calcium-activated factors are needed for the acute restriction of membrane permeability after damage, and suggesting that while ANXA7 and ANXA11 are dispensable for ESCRT-III recruitment, they are necessary for ESCRT-III turnover.

### Some pathogenic ALS-associated ANXA11 mutants are not recruited to sites of plasma membrane damage

Pathogenic mutations in ANXA11 (including G38R, R235Q, R346C, and R456H) are associated with ALS.^41^^,^^66^ A P93S mutation was recently found to be a pathogenic variant in corticobasal syndrome.^67^ Given the sequential recruitment of two FTD/ALS-associated proteins to sites of plasma membrane damage, the impaired turnover of FTD-causing C-truncated forms of CHMP2B at these damage sites, and the similarly impaired turnover of wild-type CHMP2B in cells lacking ANXA7 and ANXA11, we next asked whether any pathogenic mutations in ANXA11 also disrupted recruitment of these factors during plasma membrane repair. To test this, we examined recruitment of various forms of ANXA11-mCh to laser ablated lesions of the plasma membrane. Alongside pathogenic variants, we included an LCD deletion (ANXA11^δLCD^) and a variant of ANXA11 (ANXA11^4D/E-A^) bearing mutations in the four calcium-binding G-X-G-T-[X_38_]-[D/E] motifs.^68^ Like wild-type ANXA11-mCh, ANXA11^G38R^-mCh, ANXA11^P93S^-mCh, and ANXA11^R346C^-mCh were recruited immediately to the site of damage and concentrated rapidly at a focus that protruded from the cell (Figures S4C and S4D; Mendeley Data Videos 18–21). In contrast, ANXA11^δLCD^-mCh, ANXA11^R235Q^-mCh, ANXA11^R456H^-mCh, and ANXA11^4D/E-A^-mCh did not assemble at sites of membrane damage (Figures S4C and S4D; Mendeley Data Videos 22–25). Consistent with the calcium-dependent and 1,6-hexanediol-sensitive assembly of ANXA11, these data suggest that calcium binding and ANXA11's LCD are essential for ANXA11 clustering at sites of membrane damage. Further, these data suggest that some, but not all, ALS-associated mutations in ANXA11 impair its clustering at sites of plasma membrane damage. Consistent with the annexin-independent localization of ESCRT-III (Figures 4A and 4B), CHMP2B-L-mEGFP was recruited to all sites of damage, regardless of the expression of wild-type or mutated ANXA11-mCh (Figures S4C and S4E).

### ALS-associated ANXA11 mutants that do not assemble at sites of plasma membrane damage impair CHMP2B turnover and lead to inefficient repair

In ANXA7/ANXA11-depleted cells stably expressing small interfering RNA (siRNA)-resistant ANXA11-mCh (ANXA11-mCh^R^), ANXA11-mCh^R^ was immediately recruited to the site of damage, with CHMP2B-L-mEGFP later brought to a focus at the cell-proximal side of the ANXA11-mCh^R^-positive protrusion (Figures 4G and 4H; Video S5). In ANXA7/ANXA11-depleted cells stably expressing ANXA11^δLCD^-mCh^R^ or ANXA11^R235Q^-mCh^R^, these mutants were not assembled at the site of membrane damage (Figure 4G). While CHMP2B-L-mEGFP was still recruited on schedule, it was not focused to a point (Figure S4F) and kept accumulating, suggesting that its turnover was impaired (Figures 4G and 4H; Video S5). We used SYTOX Blue as a reporter of plasma membrane integrity and found that ANXA11^R235Q^-mCh^R^ was less able than ANXA11-mCh^R^ to rescue the persistent post-damage leakiness associated with ANXA7/ANXA11 depletion and allowed a secondary influx of PI after ablation (Figures S5A and S5B; Mendeley Data Videos 26 and 27). These data suggest that mutations impairing the ability of ANXA11 to cluster at sites of plasma membrane damage compromise the cell’s ability to make a lasting repair to the damaged membrane.

Video S5. CHMP2B-L-mEGFP turnover at sites of plasma membrane damage is impaired in ANXA7/ANXA11-depleted cells re-expressing siRNA-resistant ANXA11^δLCD^-mCh^R^ or ANXA11^R235Q^-mCh^R^, related to Figure 4 CAL-51 cells stably expressing CHMP2B-L-mEGFP and siRNA resistant ANXA11-mCh^R^, ANXA11^δLCD^-mCh^R^ or ANXA11^R235Q^-mCh^R^ were transfected with ANXA7 and ANXA11-targeting siRNA, imaged live and subject to a single pixel multi-photon laser ablation at the plasma membrane (T = 0 seconds, site marked by arrowhead). Frames were acquired every 5 seconds. Rescue with ANXA11-mCh^R^ (n = 10 videos, N = 4), rescue with ANXA11^δLCD^-mCh^R^ (n = 14 videos, N = 4), rescue with ANXA11^R235Q^-mCh^R^ (n = 11 videos, N = 4). In videos, scale bar is 5 μm.

### An ANXA11-positive electron-dense plug seals holes in the damaged plasma membrane and is subsequently extruded from cells

**Figure 5:**
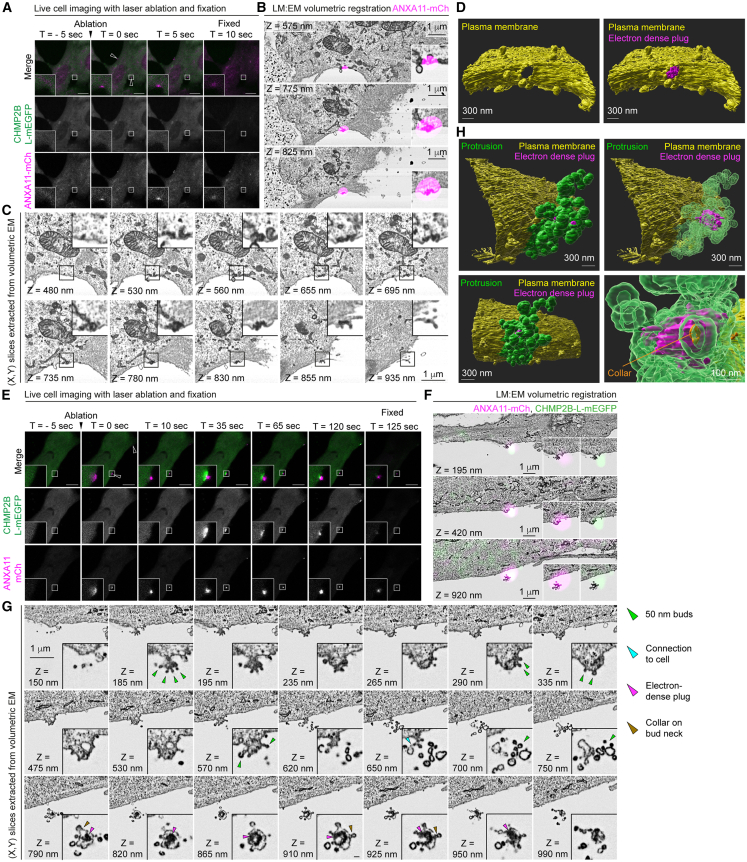
ANXA11-mCh localizes to a plug at the site of damage and is subsequently extruded from cells (A) Live imaging of cells stably expressing CHMP2B-L-mEGFP and ANXA11-mCh and fixed 5 s after laser ablation (arrowheads). Scale bar is 5 μm. (B) Overlay of co-registered correlative volumetric light microscopy and FIB-SEM images from the boxed region in Figure 5A. See also Figure S6A–S6C. (C) Select Z-slices from the FIB-SEM volume at the site of ablation. Scale bar is 1 μm and applies across all panels. (D) Volumetric EM reconstruction of the site of ablation at 5 s after ablation (from C), plasma membrane rendered in yellow, electron-dense plug in the site of ablation rendered in magenta. See also Video S6. (E) Live imaging of cells stably expressing CHMP2B-L-mEGFP and ANXA11-mCh and fixed 120 s after laser ablation (arrowheads). Scale bar is 5 μm. (F) Overlay of co-registered correlative volumetric light microscopy and FIB-SEM images from the boxed region in Figure 5E. See also Figure S6D–S6F. (G) Select Z-slices from the FIB-SEM volume at the site of ablation. Scale bar in the Z = 910 nm inset is 50 nm. Scale bar in main panels is 1 μm and applies across all panels. (H) Volumetric reconstruction of the site of ablation at 2 min after damage. Plasma membrane protrusion rendered in green and/or translucent green to allow visualization of the electron-dense plug (magenta) and collars at the bud necks (orange). See also Figure S6 and Video S6.

To examine the membrane ultrastructure at sites of plasma membrane damage, we integrated our laser-ablation assays with correlative light and electron microscopy (CLEM) and volumetric focused ion-beam scanning electron microscopy (FIB-SEM). We ablated a single pixel of the plasma membrane in cells stably expressing ANXA11-mCh and CHMP2B-L-mEGFP and fixed cells 5 s after laser ablation when ANXA11-mCh, but not CHMP2B-L-mEGFP, had been recruited to sites of damage (Figures 5A–5C and S6A–S6C; Video S6). At the ultrastructural level, an electron-dense plug was observed in a hole in the plasma membrane at the site of damage (Figure 5D; Video S6) that co-registered in 3D with the ANXA11-mCh signal (Figure 5B). At 2 min after ablation, live imaging revealed that ANXA11-mCh had formed a protrusion with CHMP2B-L-mEGFP at its base (Figure 5E; Video S6). At the ultrastructural level, our volumetric CLEM revealed that these fluorescent signals were localized to a large (approximately 500 nm in diameter) protrusion that was connected to the cell by a single convoluted membrane (Figures 5F–5H and S6D–S6G; Video S6). Importantly, this large membranous structure also contained a surface-exposed electron-dense plug (Figures 5G and 5H; Video S6). Surprisingly, the surface of this protrusion was covered in outwardly projecting 50 nm buds, and we could occasionally observe ring-like collars on their necks (Figures 5G and 5H; Video S6). Additional volumetric CLEM examples of electron-dense ANXA11-mCh positive plugs in membrane holes at 5 and 55 s after ablation are provided (Figures S6H–S6Q; Video S6). The average diameter of the electron-dense plugs was 268 ± 42 nm with a depth of 199 ± 34 nm (*N* = 4, mean ± SD). These data demonstrate the acute formation of an ANXA11-containing plug to seal the damaged membrane and suggest the subsequent removal of this plug through extrusion and ESCRT-III-dependent release.

Video S6. Correlative electron microscopy at the site of laser ablation, related to Figures 5 and S6 CAL-51 cells stably expressing ANXA11-mCh and CHMP2B-L-mEGFP were imaged live, subject to a single pixel multi-photon laser ablation at the plasma membrane, fixed on-stage at the indicated time after ablation and processed for volumetric correlative electron microscopy (vCLEM) using FIB-SEM. Concatenated video displays the correlated site of ablation at 5 seconds after damage (related to Figures 5A–5C) and a 3D reconstruction of this site with the plasma membrane illuminated in yellow and the electron dense plug illuminated in magenta (related to Figure 5D), an additional example of the correlated site of ablation fixed at 5 seconds after damage (related to Figures S6H–S6L), the correlated site of ablation fixed at 55 seconds after damage (related to Figures S6M–S6Q), the correlated site of ablation fixed at 120 seconds after damage (related to Figures 5E–5G) and a 3D reconstruction of this site with the plasma membrane illuminated in yellow, the electron dense plug illuminated in magenta, the membrane protrusion illuminated in green and collar-like structures at bud necks rendered in blue. In EM Z-stacks, voxel size is 5 nm.

### Annexins and ESCRT-III are recruited sequentially to sites of PfO-induced plasma membrane damage

**Figure 6:**
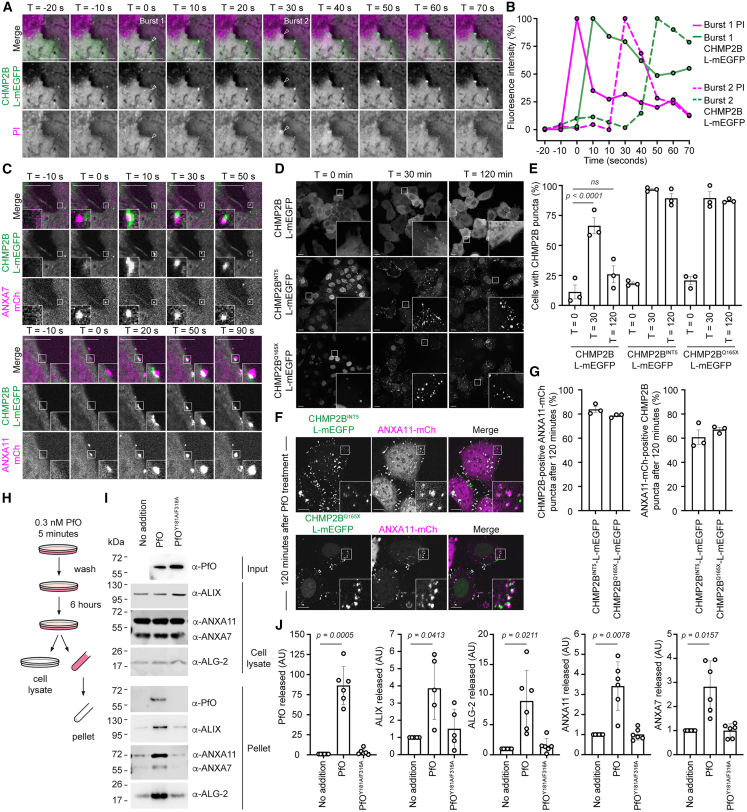
Sequential recruitment of ANXA7/ANXA11 and CHMP2B to sites of PfO-mediated membrane damage (A) Images of CAL-51 cells stably expressing CHMP2B-L-mEGFP and treated with 1 nM PfO in the presence of 160 μg/mL PI. Bursts of cytoplasmic PI entry marked with arrowheads. Scale bar is 10 μm. See corresponding Video S7. (B) Quantification of mEGFP and PI fluorescent intensities in the cytoplasmic region of Burst-1 and Burst-2 from (A) with timings relative to Burst-1 and intensities normalized by min-max scaling. (C) CAL-51 cells stably expressing CHMP2B-L-mEGFP and either ANXA7-mCh or ANXA11-mCh were treated with 1 nM PfO for 5 min, chased into fresh media and imaged live. Sequential recruitment of ANXA7-mCh/ANXA11-mCh and CHMP2B-L-mEGFP to cell-distal and cell-proximal regions was observed (ANXA7-mCh, *N* = 5) (ANXA11-mCh, *N* = 4). Timestamp set relative to annexin recruitment. Scale bar is 10 μm. See corresponding Video S7. (D and E) CAL-51 cells stably expressing CHMP2B-L-mEGFP, CHMP2B^INT5^-L-mEGFP or CHMP2B^Q165X^-L-mEGFP were treated with 1 nM PfO for 5 min and chased into fresh media. Cells displaying mEGFP puncta at the plasma membrane at the indicated times were scored manually; graph displays mean ± SEM from between 353 and 617 cells per condition analyzed across *N* = 3 independent experiments. Significance calculated by one-way ANOVA, with Tukey correction. In D, scale bar is 20 μm. (F and G) CAL-51 cells stably expressing CHMP2B^INT5^-L-mEGFP or CHMP2B^Q165X^-L-mEGFP and ANXA11-mCh were treated with 1 nM PfO for 5 min, chased into fresh media and imaged live after 120 min. (G) displays mean ± SEM (*N* = 3) of colocalization/juxtaposition of plasma membrane GFP and mCh signals from puncta in 100 cells (CHMP2B^INT5^-L-mEGFP) or 84 cells (CHMP2B^Q165X^-L-mEGFP). In (F), examples of colocalization displayed by arrowheads, scale bar is 10 μm. (H) Schematic for PfO-dependent shedding assay. (I) Resolved PfO input, cell lysates and pelleted fractions were examined by western blotting with antibodies that recognise PfO, ALIX, ANXA7, ANXA11, or ALG-2. (J) Quantification of band intensity of pellets in (I). All values were normalized to “no addition” control signal. Graphs display mean ± SD from *N* = 5 (ALIX) or *N* = 6 (all others) independent experiments with significance calculated by one-way ANOVA with Dunnett’s correction. See also Figure S7.

To analyze release of material after damage, we next generated recombinant versions of the *Clostridium perfringens* cholesterol-dependent cytolysin, perfringolysin O (PfO). Consistent with established oligomerization and pore-formation times, exposing cells to 1 nM PfO in the presence of external PI revealed pore formation after 10–15 min. As with laser ablation, PI entry after PfO treatment was transient, appearing as a burst (Figures 6A and 6B; Video S7). These data suggest that once permeant, PfO pores are rapidly sealed, presumably through an acute mechanism to restrict cytolysis. CHMP2B-L-mEGFP was recruited to sites of PfO-mediated PI influx, but only after the burst of PI had abated (Figures 6A and 6B; Video S7). These data suggest that across different insults, cells mount an acute response to restrict permeability after membrane damage and that ESCRT-III components are recruited after this restriction has been formed.

Video S7. PI entry at sites of PfO-mediated plasma membrane damage is transient with CHMP2B-L-mEGFP recruitment occurring after PI entry has been restricted and after ANXA7-mCh and ANXA11-mCh have been recruited, related to Figure 6 Concatenated videos showing CAL-51 cells stably expressing CHMP2B-L-mEGFP were treated with 1 nM PfO and imaged live in the presence of 160 μg/ml PI. Frames were acquired every 10 seconds and frames proximal to PI entry are presented. Scale bar is 10 μm. Subsequent videos show CAL-51 cells stably expressing ANXA7-mCh and CHMP2B-L-mEGFP or ANXA11-mCh and CHMP2B-L-mEGFP were treated with 1 nM PfO for 5 minutes, washed thrice, released into fresh imaging media and imaged live. Frames from N = 5 (ANXA7) or N = 4 (ANXA11) independently imaged cells were acquired every 10 seconds. Timestamp relative to appearance of ANXA-mCh puncta. Scale bar is 5 μm.

Pore forming toxins (PFTs) can be collected from the media of treated cells,^69^^,^^70^ and ESCRT-III proteins localize to the plasma membrane of cells damaged by mixed lineage kinase domain-like protein (MLKL) or gasdermin-D,^8^^,^^71^ suggesting that ESCRT-III may contribute to the release of membranes containing these agents. We verified that mutations in PfO that allowed membrane binding but prevented its cytolytic activity (PfO^Y181A/F318A^ and PfO^K336E^)^72^^,^^73^ prevented PI influx (Figures S7A–S7C) and the recruitment of CHMP2B-L-mEGFP (Figure S7D), linking CHMP2B recruitment to the pore-forming activity of the toxin. Using doubly stable cell lines, we confirmed that ANXA7-mCh or ANXA11-mCh and CHMP2B-L-mEGFP were recruited sequentially to sites of PfO-mediated membrane damage. After PfO treatment, ANXA7-mCh and ANXA11-mCh formed protrusions at the plasma membrane with CHMP2B-L-mEGFP localizing to their base (Figure 6C; Video S7). As we had observed with laser ablation, while CHMP2B-L-mEGFP assembled transiently at the plasma membrane after PfO treatment, CHMP2B^INT5^-L-mEGFP and CHMP2B^Q165X^-L-mEGFP persisted (Figures 6D, 6E, S7E, and S7F; Mendeley Data Videos 28–30). In cells treated with PfO for 5 min and imaged 2 h later, we found that the persistent CHMP2B^INT5^-L-mEGFP and CHMP2B^Q165X^-L-mEGFP puncta retained ANXA11-mCh (Figures 6F and 6G). We confirmed that endogenous ANXA11 was similarly retained at persistent CHMP2B^INT5^-L-mEGFP and CHMP2B^Q165X^-L-mEGFP puncta 2 h after PfO treatment, whereas it was largely cleared from the plasma membrane in cells expressing CHMP2B-L-mEGFP (Figures S7G and S7H). These data link impaired turnover of the C-truncated CHMP2B mutants with the retention of ANXA11 at sites of plasma membrane damage.

### Damaged membranes are shed from cells

During timelapse imaging of PfO-treated cells stably expressing CHMP2B-L-mEGFP and either ANXA7-mCh or ANXA11-mCh, we could detect the abrupt disappearance of the annexin-mCh protrusions in the frames after CHMP2B-L-mEGFP recruitment, suggesting that annexin-positive protrusions are released (Figures S7I and S7J; Mendeley Data Videos 31 and 32), paralleling observations made with laser ablation and CLEM. Turning to biochemical methods, after a sub-lytic pulse of PfO, we found that PfO, ANXA7, ANXA11, ALIX, and ALG-2 could be recovered from the media. Importantly, release of these proteins was not observed in cells treated with PfO^Y181A/F318A^ (Figures 6H–6J), linking ESCRT-III recruitment and release of these membranes directly to the membrane damaging activity of this toxin.

### ESCRT-III activity ensures the bilayer is robustly sealed

**Figure 7:**
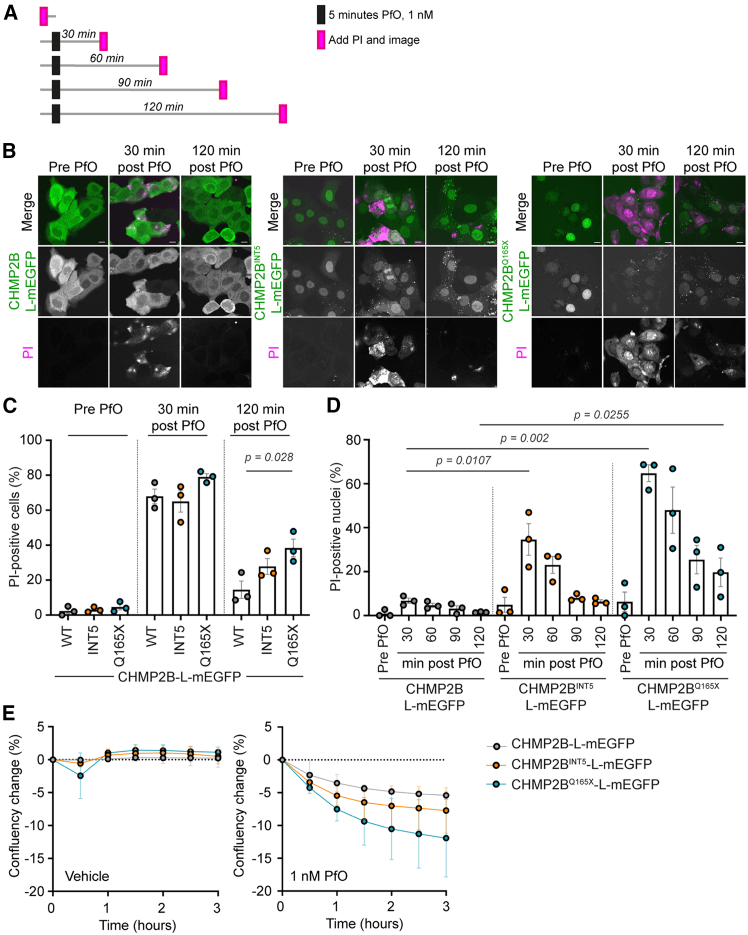
Cells bearing FTD-causing mutations in CHMP2B are more vulnerable to plasma membrane damage (A) Schematic of membrane damage protocol to examine time-dependent membrane repair. (B–D) Representative images (B) and quantification (C and D) of CAL-51 cells stably expressing CHMP2B-L-mEGFP, CHMP2B^INT5^-L-mEGFP, and CHMP2B^Q165X^-L-mEGFP, treated with 1 nM PfO for 5 min, chased into fresh media and challenged with PI at the indicated time points. Scale bar is 10 μm. The number of PI-positive cells (C) and the number of cells with PI-positive nuclei (indicative of saturation) (D) was quantified from between 418 and 666 cells per condition (C) or between 85 and 271 cells per condition (D) from *N* = 3 independent experiments in each case. Mean ± SEM displayed with statistical significance determined by unpaired two-tailed *t* test (C), or one-way ANOVA (D). (E) CAL-51 cells stably expressing CHMP2B-L-mEGFP, CHMP2B^INT5^-L-mEGFP, and CHMP2B^Q165X^-L-mEGFP were treated with the indicated concentration of PfO and imaged live using Incucyte microscopy. Cytotoxicity led to a reduced confluency of the monolayer and was calculated from *N* = 2 independent experiments with experimental means normalized to T = 0. Graphs display mean ± SD.

We next wanted to ask if ANXA7/ANXA11 assembly represented a temporary “seal” to restrict cytoplasm loss, followed by ESCRT-III-dependent shedding of this membrane to “heal” the membrane bilayer. Given the failure to release ANXA11 protrusions in cells expressing C-truncated versions of CHMP2B (Figures 6D–6G), we investigated the robustness of this seal by treating cells with a 5 min pulse of PfO and probing plasma membrane integrity at sequential times after removal of PfO (Figure 7A). At 30 min after PfO addition, CHMP2B-L-mEGFP, CHMP2B^INT5^-L-mEGFP, or CHMP2B^Q165X^-L-mEGFP cells were equally susceptible to PI challenge (Figures 7B and 7C). Allowing a chase of 120 min before challenging with external PI revealed that cells expressing CHMP2B-L-mEGFP were now impermeable to PI. However, cells expressing CHMP2B^INT5^-L-mEGFP or CHMP2B^Q165X^-L-mEGFP were still able to allow PI entry (Figures 7B and 7C), suggesting that the plasma membrane integrity of these cells was still impacted by the PfO treatment. Cells expressing CHMP2B^INT5^-L-mEGFP or CHMP2B^Q165X^-L-mEGFP showed greater PI influx over this period of chase, leading to significant nuclear accumulation (Figures 7B and 7D). We next exposed cells to PfO and followed cell death by Incucyte microscopy. We found that cells expressing CHMP2B^INT5^-L-mEGFP or CHMP2B^Q165X^-L-mEGFP were more sensitive to PfO than cells expressing CHMP2B-L-mEGFP (Figure 7E). These data suggest that while cells mount an acute response to membrane damage by mobilizing annexin proteins, this acute plug is not robust and requires ESCRT-III activity to expunge these plugs through a process of shedding. Moreover, these data suggest that cells bearing pathogenic mutations of CHMP2B are less able to maintain membrane integrity and more susceptible to cell death upon plasma membrane damage.

## Discussion

Here, we have used advanced imaging to illuminate how annexin and ESCRT-III proteins work together to repair damaged plasma membranes. Using independent forms of plasma membrane damage, we show that arrival of the calcium-binding ANXA7 or ANXA11 proteins at plasma membrane lesions occurs concomitantly with an acute restriction of plasma membrane permeability. Our volumetric CLEM suggests that these annexins form an acute plug to restrict cytolysis and facilitate subsequent ESCRT-III-dependent repair. Interestingly, given the requirement for the ANXA11 LCD and its sensitivity to 1,6-hexanediol, this plug formation has parallels with the condensation of stress granules that are proposed to seal damaged endolysosomes,^17^ the phase separation of LEMD2’s LCD that helps to seal holes in the reforming nuclear envelope,^74^ and the diffusion barriers that maintain nucleocytoplasmic compartmentalization before ESCRT-III activity during spindle pole body extrusion in *S. pombe*.^75^ Indeed, the finding that biomolecular condensates can contribute to the membrane bending necessary for intra-endosomal vesicle formation^76^ suggests that LCD-dependent condensation of annexin proteins may help to generate curved membranes for ESCRT-III to assemble upon. Given that it was possible to capture ANXA7- and ANXA11-positive membranes after damage, and by drawing analogies with ESCRT-III-dependent viral release, we suggest that ESCRT-III acts to release this protrusion, eliminating the damaged membrane and creating a scarless membrane seal.

Our volumetric CLEM revealed that the surface of the plug-containing protrusion was covered by 50 nm buds, and its connection to the cell was via a membrane tube of similar diameter. These match the dimensions of structures that ESCRT-III is known to sever and the collars around the necks of these buds were highly reminiscent of the collars observed around budding HIV-1 virions.^77^ While prior data have suggested that recruitment of ESCRT-III proteins occurred coincident with membrane sealing, we found that CHMP2B-L-mEGFP was only recruited to sites of damage after permeability had been restricted. Consistent with this, in a recent cryo-electron tomography study of perforin-mediated cytolysis, the plasma membrane appeared intact at times of ESCRT-III recruitment.^61^ CHMP2B has been shown to assemble at plasma membrane protrusions^78^ and can act as a diffusion barrier to restrict lipid flow into membrane necks pulled from giant unilamellar vesicles.^79^ We speculate that this activity might also isolate damaged membrane lipids and the annexin plug for subsequent ESCRT-III-dependent shedding.

While annexin and ESCRT-III proteins function in the same membrane repair pathway, the initial recruitment of CHMP2B to sites of damage was independent of ANXA7 and ANXA11. Indeed, while annexin recruitment was strictly calcium dependent, CHMP2B recruitment was calcium independent, suggesting an as yet unknown pathway for mobilizing ESCRT-III. While Sønder et al. have shown CHMP4B recruitment is dependent upon ANXA7,^14^ we found that CHMP2B was robustly recruited in cells lacking ANXA7 and ANXA11, or in cells lacking ANXA7 and ANXA11 and reliant upon versions of ANXA11 that could not assemble at sites of membrane damage. These data suggest that parallel pathways may recruit different ESCRT-III components. Importantly, in all cases where we abrogated ANXA7 and ANXA11 recruitment to sites of plasma membrane damage, while CHMP2B was recruited normally, its turnover was compromised, leading to its persistence and accumulation at these sites. These data mimic the phenotype of C-truncated pathogenic versions of CHMP2B and suggest that clustering of these LCD-containing annexins is necessary for ESCRT-III activity, perhaps by helping to shape the membrane into a neck for ESCRT-III-dependent severing.

Why would a cell need to separate plug formation and plug extrusion? Given the time taken to mobilize ESCRT-III, an acute plug seems a sensible way to restrict cytolysis. We note that permeability restriction was possible, albeit leaky, in the absence of ANXA7 and ANXA11, which may be due to the remaining annexins present in cells. However, this plug is likely an imperfect seal (Figure S6L), and its ESCRT-III-dependent removal will allow cells to restore a robust and scarless membrane. Our data suggest a sequential "sealing and healing" model of membrane repair, whereby annexin proteins seal the membrane by forming an acute plug, followed by ESCRT-III activity to remove the damaged membrane and provide a permanent repair.

These defects in membrane repair were uncovered through the analysis of FTD- and ALS-associated mutations in CHMP2B and ANXA11. While the endosomal phenotype of C-truncated versions of CHMP2B has been well characterized, reflective of an inability to recruit VPS4 through C-terminal MIMs,^52^^,^^53^^,^^80^ we describe here differences in the subcellular localization of these mutants that leads to a kinetic delay in their recruitment to sites of membrane damage. Further, we show that turnover and ability to release damaged membranes was impaired due to an inability to bind VPS4. We identify NES and nuclear retention sequences in CHMP2 proteins that explain their relative differences in subcellular localization, confirming localizations described recently in neurons.^81^ While cells bearing the C-truncated versions of CHMP2B were more sensitive to plasma membrane damaging agents, we hypothesize that a range of cytosolic ESCRT-III activities such as lysosomal repair and multivesicular body biogenesis are compromised in cells bearing these pathogenic mutants of CHMP2B. Increasing evidence presents FTD/ALS as a spectrum of disease, inclusive of a wide range of genetic risk factors. Thus far, there is no consensus as to an underlying mechanism supporting this relationship. Here, we have demonstrated that proteins encoded by two different FTD/ALS risk factors act sequentially in the same cytoprotective process. Moreover, pathogenic mutations in both CHMP2B and ANXA11 converge on a common phenotype of impaired ESCRT-III turnover at sites of plasma membrane damage. These data suggest that plasma membrane damage may be a potential disease mechanism in FTD/ALS.

### Limitations of the study

While we have inferred a neuroprotective role for annexin- and ESCRT-III-dependent plasma membrane repair, this mechanistic study has been performed in non-central nervous system (CNS) cell lines. Moreover, the insults used, targeted laser ablation and PFTs, were selected for their ability to deliver a clear lesion in a defined time period. How the plasma membrane of cells in the CNS is damaged, or how the cumulative effect of plasma membrane damage over the life course of an organism impacts neurological health, will require further investigation.

## Resource availability

### Lead contact

Requests for further information and resources should be directed to and will be fulfilled by the lead contact, Jeremy Carlton (jeremy.carlton@kcl.ac.uk).

### Materials availability

Plasmids generated in this study are available from the lead contact, Jeremy Carlton (jeremy.carlton@kcl.ac.uk).

### Data and code availability

All data (western blot images, light microscopy, electron microscopy data, Mendeley Data Videos 1–32, Mendeley Data Table 1, and legends for Mendeley Data materials) have been deposited in Mendeley Data at https://doi.org/10.17632/cjn4b6s94d.1 and are publicly available as of the date of publication. This paper does not report original code. Any additional information required to reanalyze the data reported in this paper is available from the lead contact upon request.

## Acknowledgments

We thank Dr. Kurt Anderson, Rocco D’Antuono, and Dr. Matt Renshaw (Crick Advanced Light Microscopy) for calibration assistance for the laser-ablation assays. We thank Prof. Mark Wallace (Dept. of chemistry, King’s College London) for advice related to pore-forming toxins. C.M.H. was supported by a Medical Research Council (MRC) Doctoral Training Programme studentship. This work was supported by the Chan Zuckerberg Initiative Neurodegeneration Challenge Network (NDCN) through Collaborative Pairs awards to J.G.C. (2022-250583) and A.M.I. (2022-250420) and a Neuroscience Collaboration Supplement (2024-349563) to J.G.C. J.G.C. is a Wellcome Trust Senior Research Fellow (224484/Z/21/Z). J.G.C. and P.C.H. are supported by the Francis Crick Institute which receives its core funding from Cancer Research UK (CC1002 and CC1076), the UK Medical Research Council (CC1002 and CC1076), and the Wellcome Trust (CC1002 and CC1076). A.M.I. is supported by the UK Dementia Research Institute (UKDRI-1203) through UK DRI Ltd., principally funded by the MRC. This research was funded in whole, or in part, by the Wellcome Trust (224484/Z/21/Z, CC1002, and CC1076). For purposes of Open Access, the authors have applied a Creative Commons Attribution (CC BY) public copyright license to any author accepted manuscript version arising from this submission.

## Author contributions

Conceptualization, C.M.H., G.P.S., A.M.I., and J.G.C.; methodology, C.M.H., G.P.S., L.C.H., P.C.H., and J.G.C.; investigation, C.M.H., G.P.S., L.C.S., and J.G.C.; writing – original draft, C.M.H. and J.G.C.; writing – review & editing, C.M.H., G.P.S., L.C.S., P.C.H., A.M.I., and J.G.C.; funding acquisition, C.M.H., A.M.I., and J.G.C.; supervision, P.C.H., A.M.I., and J.G.C.

## Declaration of interests

The authors declare no competing interests.

## STAR★Methods

### Key resources table

**Table:** 

REAGENT or RESOURCE	SOURCE	IDENTIFIER
**Antibodies**		

Anti-GFP (clones 7.1 and 13.1)	Roche	Cat# 11814460001; RRID:AB_390913
Anti-CHMP2B (3335)	BioGenes	Ghazi-Noori et al.^82^
Anti-GAPDH (clone 6C5)	Millipore	Cat# MAB374; RRID:AB_2107445
Anti-ALIX	Proteintech	Cat# 12422-1-AP; RRID:AB_2162467
Anti-ANXA7	Proteintech	Cat# 10154-2-AP; RRID:AB_2227386
Anti-ANXA11	Proteintech	Cat# 10479-2-AP; RRID:AB_2057171
Anti-ALG-2	Proteintech	Cat# 12303-1-AP; RRID:AB_2162459
Anti-Perfringolysin-O	Abcam	Cat# ab225685; RRID: AB_3740006
Anti-p44/42 MAPK	Cell Signaling Technology	Cat# 9102; RRID:AB_330744
Anti-HIS Tag (clone J099B12)	BioLegend	Cat# 652502; RRID:AB_11204427
Anti-HA Tag (HA.11, clone 16B12)	BioLegend	Cat# 901501; RRID:AB_2565006
Alexa568-conjugated goat anti-rabbit IgG	ThermoFisher Scientific	Cat# A-11011; RRID:AB_143157
HRP-conjugated goat anti-rabbit IgG	Cell Signalling Technology	Cat# 7074; RRID:AB_2099233
HRP-conjugated goat anti-mouse IgG	Cell Signalling Technology	Cat# 7076; RRID:AB_330924
IRDye 800CW-conjugated goat anti-mouse IgG	LI-COR Biosciences	Cat# 925-32210; RRID:AB_2687825
IRDye 680CW-conjugated goat anti-rabbit IgG	LI-COR Biosciences	Cat# 925-68071; RRID:AB_2721181

**Bacterial and virus strains**		

NEB® 5-alpha Competent *E. coli*	New England Biolabs	Cat# C2987H
One Shot BL21 (DE3) Chemically Competent *E. coli*	ThermoFisher Scientific	Cat# C600003

**Chemicals, peptides, and recombinant proteins**		

Propidium Iodide	Merck	Cat# P4170, CAS: 25535-16-4
SYTOX Blue	ThermoFisher Scientific	Cat# S11348
MitoTracker DeepRed	ThermoFisher Scientific	Cat# M46753
Leptomycin B	Cayman Chemicals	Cat# 10004976, CAS: 87081-35-4
Cytochalasin D	Merck	Cat# C8273, CAS: 22144-77-0
Lipofectamine RNAiMAX	ThermoFisher Scientific	Cat# 13778075
Lipofectamine-3000	ThermoFisher Scientific	Cat# L3000008
25-kDa linear polyethylenimine	Polysciences	Cat# 23966, CAS: 26913-06-4
Dulbecco’s Modified Eagle’s Medium	ThermoFisher Scientific	Cat# 41966029
FluoroBrite DMEM	ThermoFisher Scientific	Cat# A1896701
Calcium- and Phenol Red-free DMEM powder	USBiological	Cat# D9800-10A
Penicillin-Streptomycin (5,000 U/mL)	ThermoFisher Scientific	Cat# 15070063
Fetal Bovine Serum	ThermoFisher Scientific	Cat# A5256701
Methanol Free 4% formaldehyde	Cell Signaling Technology	Cat# 47746, CAS: 50-00-0
1,6-hexanediol	Merck	Cat# H11807, CAS: 629-11-8
16% formaldehyde, EM grade	TAAB	Cat# F017, CAS: 50-00-0
25% Glutaraldehyde, EM grade	TAAB	Cat# G011, CAS: 111-30-8
Thiocarbohydrazide	Merck	Cat# 223220, CAS: 2231-57-4
4% aqueous osmium tetroxide	TAAB	Cat# O012, CAS: 20816-12-0
Uranyl Acetate	TAAB	Cat# U007, CAS: 541-09-3
Potassium ferricyanide	Sigma Aldrich	Cat# 244023, CAS: 13746-66-2
Lead nitrate	Sigma Aldrich	Cat# 228621, CAS: 10099-74-8
L-Aspartic acid	Sigma Aldrich	Cat# A4534, CAS: 56-84-8
Durcupan ACM component A	Sigma Aldrich	Cat# 44611
Durcupan ACM component B	Sigma Aldrich	Cat# 44612
Durcupan ACM component C	Sigma Aldrich	Cat# 44613
Durcupan ACM component D	Sigma Aldrich	Cat# 44614
cOmplete Protease Inhibitor tablets	Roche	Cat# 5056489001
DNAse-1	VWR	Cat# A3778
Lysozyme	Merck	Cat# L6876, CAS: 9001-63-2
HisPur Ni-NTA resin	ThermoFisher Scientific	Cat# 88221

**Critical commercial assays**		

BioRad Protein Assay	BioRad	Cat# 5000001

**Deposited data**		

Original data for each figure in the manuscript	Mendeley	DOI:10.17632/cjn4b6s94d.1
Videos S1, S2, S3, S4, S5, S6, and S7 and Mendeley Data Videos 1 - 32	Mendeley	DOI:10.17632/cjn4b6s94d.1
Mendeley Data Table 1	Mendeley	DOI:10.17632/cjn4b6s94d.1
Legends for Mendeley Data Videos 1 - 32 and Mendeley Data Table 1	Mendeley	DOI:10.17632/cjn4b6s94d.1

**Experimental models: Cell lines**		

Human Hek293 derived GP2-293 cells	Francis Crick Institute Cell Science platform	RRID:CVCL_WI48
Human Hek293T derived Phoenix-AMPHO cells	Francis Crick Institute Cell Science platform	RRID:CVCL_H716
Human CAL-51 cells	Francis Crick Institute Cell Science platform	RRID:CVCL_1110

**Oligonucleotides**		

Control siRNA	Horizon Discovery	D-001810-01
ANXA7 siRNA: CCGAGAAAUUGUCAGAUGU	Horizon Discovery	J-010760-08
ANXA11 siRNA: GAAGAUCUGUGGUGGCAAU	Horizon Discovery	J-011212-05
gRNA targeting CHMP2B C terminus: AGAGATTGAACGGCAACTCA	Integrated DNA Technology	NA
gRNA targeting CHMP2B Intron5 junction: GAACTTGATTCACAATATCC	Integrated DNA Technology	NA

**Recombinant DNA**		

pCMS28	Carlton and Martin-Serrano^83^	N/A
pCMS28 CHMP2B-L-mEGFP	This study	N/A
pCMS28 CHMP2B^INT5^-L-mEGFP	This study	N/A
pCMS28 CHMP2B^Q165X^-L-mEGFP	This study	N/A
pCMS28 CHMP2B^INT5:c7α6^-L-mEGFP	This study	N/A
pCMS28 CHMP2B^Q165X:c7α6^-L-mEGFP	This study	N/A
pCMS28 CHMP2B^INT5:c7α6(ES-RR)^-L-mEGFP	This study	N/A
pCMS28 CHMP2B^L167A/I170A/I172A^-L-mEGFP	This study	N/A
pCMS28 CHMP2B^I203A/L207A/L210A/V212A^-L-mEGFP	This study	N/A
pCMS28 CHMP2B^V212K^-L-mEGFP	This study	N/A
pCMS28 CHMP2B^K59A/K63A/H67A/K70A/R74A^-L-mEGFP	This study	N/A
pCMS28 CHMP2B^INT5:K59A/K63A/H67A/K70A/R74A^-L-mEGFP	This study	N/A
pCR3.1 CHMP2B-HA	This study	N/A
pCR3.1 CHMP2B^INT5^-HA	This study	N/A
pCR3.1 CHMP2B^Q165X^-HA	This study	N/A
pSniperCas9-CHMP2B^CT^-P2A-tagBFP	This study	N/A
pSniperCas9-CHMP2B^INT5^-P2A-tagBFP	This study	N/A
pGW57-AMP CHMP2B-L-mClover3 HDR repair	This study	N/A
pGW57-AMP CHMP2B^INT5^-L-mClover3 HDR repair	This study	N/A
pCMS28 CHMP2A	This study	N/A
pCMS28 CHMP2A^c7α6^-L-mEGFP	This study	N/A
pCMS28 CHMP2A^R221L^-L-mEGFP	This study	N/A
pCMS28 CHMP2A^R60A/K64A/R68A/R71A/K75A^-L-mEGFP	This study	N/A
pCR3.1 mEGFP	This study	N/A
pCR3.1 YFP-CHMP2A	Olmos et al.^56^	N/A
pCR3.1 YFP-CHMP2A^1-54^	Olmos et al.^56^	N/A
pCR3.1 YFP-CHMP2A^1-64^	Olmos et al.^56^	N/A
pCR3.1 YFP-CHMP2A^1-74^	Olmos et al.^56^	N/A
pCR3.1 YFP-CHMP2A^1-84^	Olmos et al.^56^	N/A
pCR3.1 YFP-CHMP2A^1-94^	Olmos et al.^56^	N/A
pCR3.1 YFP-CHMP2A^1-104^	Olmos et al.^56^	N/A
pCR3.1 YFP-CHMP2A^1-117^	Olmos et al.^56^	N/A
pCR3.1 YFP-CHMP2A^1-139^	Olmos et al.^56^	N/A
pCR3.1 YFP-CHMP2A^1-146^	Olmos et al.^56^	N/A
pCR3.1 YFP-CHMP2A^1-154^	Olmos et al.^56^	N/A
pCR3.1 YFP-CHMP2A^1-160^	Olmos et al.^56^	N/A
pCR3.1 YFP-CHMP2A^1-172^	Olmos et al.^56^	N/A
pCR3.1 YFP-CHMP2A^1-177^	Olmos et al.^56^	N/A
pMSCVneo ANXA7-mCherry	This study	N/A
pMSCVneo ANXA11-mCherry	This study	N/A
pMSCVneo ANXA11^dLCD^-mCherry	This study	N/A
pMSCVneo ANXA11^G38R^-mCherry	This study	N/A
pMSCVneo ANXA11^P93S^-mCherry	This study	N/A
pMSCVneo ANXA11^R235Q^-mCherry	This study	N/A
pMSCVneo ANXA11^R346C^-mCherry	This study	N/A
pMSCVneo ANXA11^R456H^-mCherry	This study	N/A
pMSCVneo ANXA11^E257A/D329A/E413A/D488A^-mCherry	This study	N/A
pMSCVneo ALG-2-mCherry	This study	N/A
pGFP-CHMP2A^56^^-82^-β-Galactosidase	This study	N/A
pGFP-CHMP2B^55^^-81^-β-Galactosidase	This study	N/A
pLAMP1-tdTomato	Max Gutierrez, Francis Crick Institute, London	N/A
pCR3.1 mCherry-PLCδ1-PH	This study	N/A
C1-MPAct-mRuby3 (MPAct-mRuby3)	Tobias Meyer, Weill Cornell, New York	RRID:Addgene_155221
pcDNA-Ran-T24N-mRFP1-polyA (RAN^T24N^-mRFP1)	Yi Zhang, HHMI, Boston, Massachusetts	RRID:Addgene_104561
pcDNA-Ran-Q69L-mRFP1-polyA (RAN^Q69L^-mRFP1)	Yi Zhang, HHMI, Boston, Massachusetts	RRID:Addgene_104560
pHM830 (GFP-β-Galactosidase)	Thomas Stamminger, Ulm University	RRID:Addgene_20702
pHM840 (GFP-NLS^SV40^-β-Galactosidase)	Thomas Stamminger, Ulm University	RRID:Addgene_20701
pET28a	Novagen	Cat# 69864-M
pPFO	Alejandro Heuck, UMass, Amherst	N/A

**Software and algorithms**		

ImageJ/FIJI	Rueden et al.^84^	https://imagej.net/software/fiji/downloads
AMST2	Hennies et al.^85^	https://github.com/jhennies/AMST2
BigWARP	Bogovic et al.^86^	https://imagej.net/plugins/bigwarp
TrakEM2	Cardona et al.^87^	https://imagej.net/plugins/trakem2/
Imaris v11	NA	https://imaris.oxinst.com/

**Other**		

8-well μslides	ibidi	Cat# 80826
35 mm Dish | No. 1.5 Gridded Coverslip	MatTek	Cat# P35G-1.5-14-C-GRD
Phenoplate optically clear Poly D Lysine-coated 96-well plates	Revvity	Cat# 6055500

### Experimental Model and Study Participant Details

#### Cell culture

GP2-293 (CVCL_WI48), Phoenix-AMPHO (CVCL_H716) and CAL-51 (CVCL_1110) cells were obtained from the Crick Cell Services Science Technology Platform. CAL-51 cells are a female diploid human breast cancer line with a stable karyotype. GP2-293 and Phoenix-AMPHO cells are derived from Hek293 cells (CVCL_0045), a transformed human female fetal cell line. The influence of sex on the results of the study was not considered. Cell lines were authenticated by Short-Tandem Repeat profiling and were validated to be free of mycoplasma by the Francis Crick Institute Cell Science Platform. No ethical approval is required for the use of these cell lines. Cells were cultured at 37 °C and at 5% CO_2_ in Dulbecco’s Modified Eagle Medium (DMEM) containing 10% FBS, penicillin (100 U/mL) and streptomycin (0.1 mg/mL). For live cell imaging, DMEM was exchanged for FluoroBrite DMEM (Gibco) with identical supplementation and 2 mM L-glutamine. Calcium- and Phenol Red-free DMEM powder was from USBiological (D9800-10A). 1,6-hexanediol (H11807) was from Sigma and was reconstituted in FluoroBrite DMEM.

#### Microbe strains

*Escherichia coli* (*E. coli*) strains DH5α (NEB) and BL21(DE3) (ThermoFisher Scientific) were used for cloning and protein expression respectively.

### Method details

#### CRISPR/Cas9-mediated editing

Guide RNAs targeting the C terminus of CHMP2B (AGAGATTGAACGGCAACTCA) or the C terminus of CHMP2B Exon-5 (GAACTTGATTCACAATATCC) were cloned into pX330-SniperCas9-tagBFP^88^ and transfected into CAL-51 cells alongside HDR-repair templates containing 800 nucleotides of homology arms flanking the L-mClover3 sequence at the relevant C terminus. mClover3^+^ cells were sorted by Fluorescence-Activated Cell Sorting and clones were validated by western blotting.

#### Plasmids

cDNAs encoding coding sequences for CHMP2B, CHMP2B^INT5^ or CHMP2B^Q165X^ were synthesised with flanking 5’ *EcoRI* and 3’ *NotI* sites and cloned into the *EcoRI* and *NotI* sites of a modified version of the retroviral expression vector pCMS28^83^ bearing a mEGFP-based Localisation and Affinity Purification tag^50^ known to preserve the function of ESCRT-III proteins.^89^ This created the vector series pCMS28 CHMP2B-L-mEGFP, pCMS28 CHMP2B^INT5^-L-mEGFP and pCMS28 CHMP2B^Q165X^-L-mEGFP. CHMP2B coding sequences were transferred to pCR3.1 *EcoRI*-*XhoI*-*NotI*-HA by subcloning. Codon optimised, RNAi-resistant coding sequences for ANXA7 and ANXA11 were synthesised (Integrated DNA Technology) with 5’ *EcoRI* and 3’ *NotI* sites and cloned into the *EcoRI* and *NotI* sites of a modified version of pMSCVneo bearing an mCherry sequence C-terminal to the *NotI* site. A plasmid encoding Perfringolysin-O (PfO) was a kind gift from Prof. Alejandro Heuck (Univ. Massachusetts, Amherst). PfO sequences were amplified with flanking *EcoRI* and *NotI* sites and cloned into the *EcoRI* and *NotI* sites of pET28a (Novagen). Plasmids encoding GFP-β-Galactosidase (pHM830) and GFP-NLS^SV40^-β-Galactosidase (pHM840) were kind gifts from Prof. Thomas Stamminger (Ulm University Medical Center) via Addgene (20702 and 20701). The indicated sequences from CHMP2A and CHMP2B were cloned into the *NheI* and *AgeI* sites of pHM830. Plasmids encoding RAN^T24N^-mRFP1 and RAN^Q69L^-mRFP1 were kind gifts from Prof. Yi Zhang (Harvard Medical School) via Addgene (104561 and 104560). A plasmid encoding MP-Act-mRuby3 was a kind gift from Prof Tobias Meyer (Stanford) via Addgene (155221). A plasmid encoding LAMP1-tdTomato was a kind gift from Dr Max Gutierrez (Francis Crick Institute). Chimeric coding sequences were synthesised as gBlock dsDNA fragments (Integrated DNA Technology) with flanking *EcoRI* and *NotI* sites and cloned into expression vectors as needed. Mutations were created by standard Polymerase Chain Reaction-based techniques. Plasmids were verified by Sanger sequencing (GeneWIZ).

#### Antibodies and chemical reagents

Anti-GFP (clone 7.1/13.1) was from Roche; in-house anti-CHMP2B (3335) was developed by BioGenes as previously described^82^; anti-GAPDH (clone 6C5, MAB374) was from Millipore; anti-ALIX (12422-1-AP), anti-ANXA7 (10154-2-AP), anti-ANXA11 (10479-2-AP) and anti-ALG-2 (12303-1-AP) were from Proteintech; anti-PfO (ab225685) was from Abcam; anti-p42/p44 MAPK (9102) was from Cell Signalling Technology; anti-HIS (652502, clone J099B12) was from BioLegend; anti-HA.11 (clone 16B12) was from BioLegend. Alexa^568^-conjugated anti-rabbit secondary was from ThermoFisher (A-11011), HRP-conjugated secondary antibodies (7074 and 7076) were from Cell Signalling Technology. IRDye 800 CW (925-32210) and IRDye 680 RD (925-68071) were from LI-COR Biosciences. Propidium Iodide was from Merck. SytoxBlue was from ThermoFisher. Leptomycin B was from Cayman Chemicals. Cytochalasin D was from Merck.

#### Transient transfection of cDNA

CAL-51 cells were transfected using Lipofectamine-3000 (Life Technologies) according to the manufacturer’s instructions. GP2-293 or Phoenix-AMPHO cells were transfected using linear 25-kDa polyethylenimine (PEI, Polysciences, Inc.), as described previously.^83^

#### siRNA depletion

All siRNA-based depletions were performed at 20 nM final concentration using Lipofectamine RNAiMAX (Life Technologies) according to the manufacturer’s instructions. siRNA were obtained from Horizon Discovery. Control siRNA was D-001810-01. ANXA7 was depleted with J-010760-08 (CCGAGAAAUUGUCAGAUGU), ANXA11 was depleted with J-011212-05 (GAAGAUCUGUGGUGGCAAU).

#### Retrovirus generation

GP2-293 or Pheonix-AMPHO cells were transfected with retroviral expression vectors and pVSVG (Takara). Viral supernatants were harvested 48 hours later, purified by low-speed centrifugation and 0.2 μm syringe filtration and added to target cells in the presence of 0.8 μg/mL polybrene. Selective antibiotics (Puromycin, 1 μg/mL; G418 0.8 mg/mL) were applied after 48 hours. The degree of overexpression of GFP-tagged CHMP2B relative to endogenous CHMP2B is: CHMP2B-L-mEGFP, 3.7x; CHMP2B^INT5^-L-mEGFP, 2.1x; CHMP2B^Q165X^-L-mEGFP, 1.5x, calculated by densitometry from Figure 1A.

#### Fixation and immunofluorescence

Cells were plated on 13 mm #1.5 coverslips and fixed in Methanol-free 4 % formaldehyde(Cell Signaling Technology) for 20 minutes at room temperature, then washed thrice in PBS. For staining, fixed cells were permeabilized using 0.2 % Triton-X100, washed thrice in PBS, quenched with 0.2 M glycine and blocked in 1 % BSA for 30 minutes. Cells were incubated with primary antibodies (1:200) in 1 % BSA for 2 hours at room temperature, washed thrice with PBS before incubation with secondary antibodies in 1 % BSA for 1 hour at room temperature. Coverslips were washed and mounted onto microscope slides with fluorescent mounting medium (Dako) and imaged using an Andor Dragonfly 200 spinning disc confocal paired with a Zyla 5.5 sCMOS camera and using a Nikon Eclipse Ti2 with Plan Apo 60x/1.4NA or 100x/1.45NA objectives.

#### Live cell imaging and laser ablation microscopy

Cells expressing the indicated proteins were plated in 8-well μslides (Ibidi) approximately 24 hours prior to imaging. Live imaging was performed using the Andor Dragonfly 200 spinning disc confocal, as described above, with an environmental chamber (Okolabs) to maintain environmental conditions at 37 °C and 5% CO_2_. For laser ablation assays cells were imaged using a Zeiss LSM 880 Multiphoton with Delay Line imaging system enclosed in a Tokai Hit stage top incubator at 37 ^o^C, 5 % CO_2_. Images were acquired with a 60X oil immersion objective every 5 s. After the first 5 frames, cells were ablated at a single pixel (0.09 μm^2^) with a Chameleon multi-photon near-infrared pulsed laser (Coherent) tuned at 800 nm, typically with 15-20 % laser power. At the beginning of each imaging experiment, a test ablation was performed to ensure optimal membrane targeting. The collimator position was then adjusted to alter the z-plane as required. In experiments investigating membrane permeability, cells were preincubated for 10 minutes with either propidium iodide (PI) (160 μg/mL) or Sytox Blue (1 μg/mL) in FluoroBrite DMEM as indicated.

#### Correlative light and electron microscopy

Cells were imaged live and ablated as described above, excepting that the media was supplemented additionally with Mitotracker Deep Red FM (100 nM) and cells were imaged on grid-etched glass-bottomed 35 mm dishes (P35G-1.5-14-C-GRD, MatTek) in 2 mL of Fluorobrite DMEM and with the lid removed. After ablation, cells were fixed at an appropriate time on stage at 37 ^o^C through addition of 2 mL of prewarmed 8% formaldehyde (FA) (TAAB) in 0.2 M Phosphate Buffer (PB), pH 7.4, diluting the fixative to a final working concentration of 4% FA in 0.1 M PB, and left for 15 min. A multi-channel post fixation Z-stack was acquired. The initial fixative was replaced with EM fixative with glutaraldehyde (GA) (TAAB) (2.5% GA + 4% FA in 0.1 M PB at RT) for 30 min before further processing in the Pelco BioWave Pro+ microwave (Ted Pella) with a SteadyTemp plate set to 21 °C unless otherwise stated. All wash cycles were of 2 x 1 minute at RT and 2 x 40 seconds in the BioWave at 250W. Briefly, samples were washed in 0.1M PB before post fixation in 2% reduced osmium (TAAB) in 1.5% (v/v) potassium ferricyanide (Sigma) for 14 min in the BioWave under vacuum, alternating between 100W power on and off for 2 minutes each. Following another wash cycle in ddH_2_0, samples were incubated in 0.22 μm filtered 1% thiocarbohydrazide (Sigma) for 14 min in the BioWave under vacuum and with the SteadyTemp plate at 40 °C. Samples were washed in ddH_2_0 again as above, then incubated in 2% aqueous OsO_4_ (TAAB) using the BioWave programming as described above. After another ddH_2_0 wash cycle, samples were taken through further BioWave staining cycles in 1% uranyl acetate (Agar Scientific) under vacuum and with the SteadyTemp plate at 40 °C, followed by 0.22 μm filtered Walton’s lead aspartate (Lead nitrate and Aspartic Acid from Sigma) at pH 5.5, under vacuum and with the SteadyTemp plate at 50 °C, with a ddH_2_0 wash cycle in between with the SteadyTemp plate set at 40 °C. Samples were dehydrated through a series of ethanol concentrations; 2 washes of 70 %, 2 x 90 % and 2 x 100 % in the BioWave for 40 seconds at 250 W each and without vacuum. Infiltration with Durcupan ACM resin (Sigma) started with 50:50 ethanol:Durcupan resin in the BioWave for 3 min at 250 W, followed by 4 x 3-minute resin changes at 250 W, all with vacuum cycling on and off every 30 seconds. Finally, samples were polymerised in a 60 °C oven for ∼48 hr. A detailed protocol can be found in Mendeley Data Table 1.

#### Focused ion beam-scanning electron microscopy (FIB-SEM)

Regions of interest (ROI) were chosen in the fluorescence images and subsequently identified in the resin blocks using the imprinted MatTek dish grid. SEM images were captured at 0° tilt at 10kV and overlaid onto the fluorescence images using Photoshop CS5, to ensure the ROI was correctly localised before FIB-SEM acquisition. Electron microscope imaging and ion beam milling were carried out using a Zeiss Crossbeam 540 Focused Ion Beam SEM with InLens backscattered electron detector and Atlas 5 software. After deposition of platinum and carbon pads to protect the sample from charging, images were acquired at 1.5 kV accelerating voltage, 0.7 nA current between FIB milling steps at 30 kV accelerating voltage, 700 pA current, using 10 or 13 μs dwell time at 5 nm isotropic resolution, over 30 μm total depth.

#### FIB-SEM dataset alignment and image processing

Pre-aligned TIFF image stacks were exported from the FIB-SEM and contrast normalised using the Enhance Contrast function in ImageJ/FIJI.^84^ Stacks were downscaled to 8-bit to reduce compute requirements. Fine alignment was carried out using the Python script AMST2.^85^^,^^90^^,^^91^^,^^92^ Sharpness and smoothness were adjusted with a combination of small pixel radius Gaussian blur (1.0 pixel) and 1–2 iterations of pixel sharpness on long and short radius (15 and 5 pixels, respectively).

#### Correlative light-electron microscopy (CLEM)

ImageJ/FIJI plugins BigWarp^86^ and TrakEM2^87^ were used for registration of the FIB-SEM and LM datasets and subsequent segmentation. Registration was performed by aligning MitoTracker and mitochondria in LM and EM datasets. Area masks from TrakEM2 were reconstructed in Imaris for visualisation.

#### Recombinant protein production

*E. coli* BL21(DE3) cells expressing pET28a-PfO plasmids were grown at 37 °C to O.D. 0.6 in LB medium, induced with 0.25 mM IPTG for 3 hours, harvested by centrifugation and resuspended in TNG500 lysis buffer (50 mM Tris-HCl pH 7.5, 500 mM NaCl, 10% w/v Glycerol) supplemented with cOmplete Protease Inhibitor tablets (Roche), 10 mM imidazole, 5 mM β-Mercaptoethanol, 0.2% Triton X-100, 1 mM PMSF, 10 μg/mL DNaseI, and 1 mg/mL lysozyme. Resuspended cells were lysed by sonication and clarified by centrifugation at 40,000 x g for 60 minutes. Supernatants were incubated with Ni-NTA resin for 2 hours at 4 °C. The resin was washed sequentially with TNG500 lysis buffer, then with TNG500 lysis buffer containing 0.01% Triton X-100 and four steps of this buffer containing increasing imidazole concentrations (20, 30, 40, 50 mM imidazole) with corresponding reductions in NaCl concentrations to preserve osmolarity. Proteins were eluted in elution buffer (50 mM Tris-HCl pH 7.5, 100 mM NaCl, 400 mM imidazole, 5 mM β-Mercaptoethanol, 10% w/v Glycerol, 0.01% Triton X-100), dialysed into TNG150 (50 mM Tris-HCl pH 7.5, 500 mM NaCl, 10% w/v Glycerol) and assessed by SDS-PAGE and western blotting to assess purity. Bio-Rad Protein Assay was used to assess concentration.

#### Incucyte microscopy

CAL-51 cells stably expressing CHMP2B-L-mEGFP, CHMP2B^INT5^-L-mEGFP or CHMP2B^Q165X^-L-mEGFP were seeded in Phenoplate 96-well plates (Revvity). PfO (1 nM) was diluted in complete imaging media and imaged using a Sartorius Incucyte SX5 at 37 °C and 5% CO_2_, capturing 9 images per well every 30 minutes. Sartorius Incucyte Analysis software was used to calculate the average confluency in each well normalised to T = 0.

#### Collection of released material by ultracentrifugation

CAL-51 cells stably expressing the indicated proteins were grown in two 15 cm dishes per condition in complete DMEM. When confluent, cells were washed thrice with PBS and then into DMEM containing 1% FBS. PfO was added at a final concentration of 0.3 nM in warmed DMEM containing 1% FBS for 10 minutes. PfO-containing media was removed, the monolayer was washed thrice with PBS, taking care to remove all residual PBS, and cells were incubated with 18 mL of DMEM containing 1% FBS. Cells were left at 37 °C for 6 hours. The media was collected and subject to low-speed centrifugation (300 x g, 5 minutes) to pellet any insoluble material and was filtered through a 5 μm PluriStrainer. 36 mL of media per condition was subject to ultracentrifugation at 100,000 x g for 6 hours at 4 °C in a SW-32-Ti swinging bucket rotor (Beckman). The media was decanted and 500 μL PBS was added to the pellet and left to resuspend overnight at 4 °C. The next morning, the pellet was resuspended and re-centrifuged at 100,000 x g in low-bind 1.5 mL ultracentrifuge tubes (Beckman) using a TLA55 fixed angle rotor (Beckman). The pellet was resuspended in 50 μL of PBS and denatured through the addition of 50 μL 2 x LDS sample buffer containing 100 mM DTT.

### Quantification and Statistical Analysis

#### Laser ablation quantification

Quantification of laser ablation timelapse imaging was performed in Fiji. For quantification of fluorescent protein recruitment, regions of interest (ROI) were drawn around the maximal zone of recruitment at the damaged membrane and projected across time points. Mean intensity values were measured in these ROIs for each frame. Corresponding mean intensity background values (from a non-ablated area of equal size ROI) were measured similarly and used for frame-wise background correction by subtraction. The baseline (average corrected intensity in the pre-ablation frames) was then subtracted from each value. For PI or SYTOX Blue measurements, a region of interest was drawn around the local area of influx at T = 0 and integrated density (Figures 2B–2D and 3E) or mean intensity (Figures 3H, S4B, and S5B) was measured across all frames. These data were normalized by subtraction (Figures 2B–2D and 3E) or division (Figures 3H, S4B, and S5B) of the pre-ablation baseline average. Data showing F/Fmax is normalized by experimental maximum expressed as a fraction of 1. Videos with significant drift, retraction of cells after membrane damage, or death were not analysed or analysis was truncated.

#### Statistical analysis

Two-tailed Student’s T-tests, or ordinary one-way ANOVA with the indicated post-hoc tests, were used to assess significance between test samples and controls or significance in response over time, as stated in figure legends. No statistical methods were used to calculate sample size, the experiments were not randomised and the investigators were not blinded to allocation during experiments and outcome assessment. Live cell imaging traces in which analysis of the behaviour could not be measured (e.g., due to drift or defocussing) were excluded. N-numbers are given as the number of independent experiments; n-numbers are given as the number of cells or images analysed across independent experiments. Details of replication and statistical tests are provided in each figure legend and were performed on independent experimental means, unless otherwise stated.

## Supplemental information

Document S1. Figures S1–S7

Document S2. Article plus supplemental information

## References

[1] Zhen Y., Radulovic M., Vietri M., Stenmark H. (2021). Sealing holes in cellular membranes. EMBO J..

[2] Barisch C., Holthuis J.C.M., Cosentino K. (2023). Membrane damage and repair: a thin line between life and death. Biol. Chem..

[3] Leung C., Hodel A.W., Brennan A.J., Lukoyanova N., Tran S., House C.M., Kondos S.C., Whisstock J.C., Dunstone M.A., Trapani J.A. (2017). Real-time visualization of perforin nanopore assembly. Nat. Nanotechnol..

[4] Aglietti R.A., Estevez A., Gupta A., Ramirez M.G., Liu P.S., Kayagaki N., Ciferri C., Dixit V.M., Dueber E.C. (2016). GsdmD p30 elicited by caspase-11 during pyroptosis forms pores in membranes. Proc. Natl. Acad. Sci. USA.

[5] Ding J., Wang K., Liu W., She Y., Sun Q., Shi J., Sun H., Wang D.-C., Shao F. (2016). Pore-forming activity and structural autoinhibition of the gasdermin family. Nature.

[6] Liu X., Zhang Z., Ruan J., Pan Y., Magupalli V.G., Wu H., Lieberman J. (2016). Inflammasome-activated gasdermin D causes pyroptosis by forming membrane pores. Nature.

[7] Sborgi L., Rühl S., Mulvihill E., Pipercevic J., Heilig R., Stahlberg H., Farady C.J., Müller D.J., Broz P., Hiller S. (2016). GSDMD membrane pore formation constitutes the mechanism of pyroptotic cell death. EMBO J..

[8] Rühl S., Shkarina K., Demarco B., Heilig R., Santos J.C., Broz P. (2018). ESCRT-dependent membrane repair negatively regulates pyroptosis downstream of GSDMD activation. Science.

[9] Kayagaki N., Kornfeld O.S., Lee B.L., Stowe I.B., O’Rourke K., Li Q., Sandoval W., Yan D., Kang J., Xu M. (2021). NINJ1 mediates plasma membrane rupture during lytic cell death. Nature.

[10] David L., Borges J.P., Hollingsworth L.R., Volchuk A., Jansen I., Garlick E., Steinberg B.E., Wu H. (2024). NINJ1 mediates plasma membrane rupture by cutting and releasing membrane disks. Cell.

[11] Menny A., Serna M., Boyd C.M., Gardner S., Joseph A.P., Morgan B.P., Topf M., Brooks N.J., Bubeck D. (2018). CryoEM reveals how the complement membrane attack complex ruptures lipid bilayers. Nat. Commun..

[12] Jimenez A.J., Maiuri P., Lafaurie-Janvore J., Divoux S., Piel M., Perez F. (2014). ESCRT Machinery Is Required for Plasma Membrane Repair. Science.

[13] Scheffer L.L., Sreetama S.C., Sharma N., Medikayala S., Brown K.J., Defour A., Jaiswal J.K. (2014). Mechanism of Ca2+-triggered ESCRT assembly and regulation of cell membrane repair. Nat. Commun..

[14] Sønder S.L., Boye T.L., Tölle R., Dengjel J., Maeda K., Jäättelä M., Simonsen A.C., Jaiswal J.K., Nylandsted J. (2019). Annexin A7 is required for ESCRT III-mediated plasma membrane repair. Sci. Rep..

[15] Williams J.K., Ngo J.M., Lehman I.M., Schekman R. (2023). Annexin A6 mediates calcium-dependent exosome secretion during plasma membrane repair. eLife.

[16] Williams J.K., Ngo J.M., Murugupandiyan A., Croall D.E., Hartzell H.C., Schekman R. (2025). Calpains orchestrate secretion of annexin-containing microvesicles during membrane repair. J. Cell Biol..

[17] Bussi C., Mangiarotti A., Vanhille-Campos C., Aylan B., Pellegrino E., Athanasiadi N., Fearns A., Rodgers A., Franzmann T.M., Šarić A. (2023). Stress granules plug and stabilize damaged endolysosomal membranes. Nature.

[18] Andrews N.W., Almeida P.E., Corrotte M. (2014). Damage control: cellular mechanisms of plasma membrane repair. Trends Cell Biol..

[19] Raj N., Weiß M.S., Vos B.E., Weischer S., Brinkmann F., Betz T., Trappmann B., Gerke V. (2024). Membrane Tension Regulation is Required for Wound Repair. Adv. Sci. (Weinh).

[20] Stefani C., Bruchez A.M., Rosasco M.G., Yoshida A.E., Fasano K.J., Levan P.F., Lorant A., Hubbard N.W., Oberst A., Stuart L.M. (2024). LITAF protects against pore-forming protein–induced cell death by promoting membrane repair. Sci. Immunol..

[21] Idone V., Tam C., Goss J.W., Toomre D., Pypaert M., Andrews N.W. (2008). Repair of injured plasma membrane by rapid Ca2+-dependent endocytosis. J. Cell Biol..

[22] Prislusky M.I., Lam J.G.T., Contreras V.R., Ng M., Chamberlain M., Pathak-Sharma S., Fields M., Zhang X., Amer A.O., Seveau S. (2024). The septin cytoskeleton is required for plasma membrane repair. EMBO Rep..

[23] Skowyra M.L., Schlesinger P.H., Naismith T.V., Hanson P.I. (2018). Triggered recruitment of ESCRT machinery promotes endolysosomal repair. Science.

[24] Radulovic M., Schink K.O., Wenzel E.M., Nähse V., Bongiovanni A., Lafont F., Stenmark H. (2018). ESCRT-mediated lysosome repair precedes lysophagy and promotes cell survival. EMBO J..

[25] Radulovic M., Wenzel E.M., Gilani S., Holland L.K., Lystad A.H., Phuyal S., Olkkonen V.M., Brech A., Jäättelä M., Maeda K. (2022). Cholesterol transfer via endoplasmic reticulum contacts mediates lysosome damage repair. EMBO J..

[26] Vietri M., Radulovic M., Stenmark H. (2020). The many functions of ESCRTs. Nat. Rev. Mol. Cell Biol..

[27] Schöneberg J., Lee I.-H., Iwasa J.H., Hurley J.H. (2017). Reverse-topology membrane scission by the ESCRT proteins. Nat. Rev. Mol. Cell Biol..

[28] Burigotto M., Carlton J.G. (2025). ESCRT-III function in membrane fission and repair. Nat. Rev. Mol. Cell Biol..

[29] Skibinski G., Parkinson N.J., Brown J.M., Chakrabarti L., Lloyd S.L., Hummerich H., Nielsen J.E., Hodges J.R., Spillantini M.G., Thusgaard T. (2005). Mutations in the endosomal ESCRTIII-complex subunit CHMP2B in frontotemporal dementia. Nat. Genet..

[30] Isaacs A.M., Johannsen P., Holm I., Nielsen J.E., FReJA consortium (2011). Frontotemporal dementia caused by CHMP2B mutations. Curr. Alzheimer Res..

[31] van der Zee J., Urwin H., Engelborghs S., Bruyland M., Vandenberghe R., Dermaut B., De Pooter T., Peeters K., Santens P., De Deyn P.P. (2008). CHMP2B C-truncating mutations in frontotemporal lobar degeneration are associated with an aberrant endosomal phenotype in vitro. Hum. Mol. Genet..

[32] Coyne A.N., Rothstein J.D. (2021). The ESCRT-III protein VPS4, but not CHMP4B or CHMP2B, is pathologically increased in familial and sporadic ALS neuronal nuclei. Acta Neuropathol. Commun..

[33] Keeley O., Coyne A.N. (2024). Nuclear and degradative functions of the ESCRT-III pathway: implications for neurodegenerative disease. Nucleus.

[34] Rodger C., Flex E., Allison R.J., Sanchis-Juan A., Hasenahuer M.A., Cecchetti S., French C.E., Edgar J.R., Carpentieri G., Ciolfi A. (2020). De Novo VPS4A Mutations Cause Multisystem Disease with Abnormal Neurodevelopment. Am. J. Hum. Genet..

[35] Coyne A.N., Baskerville V., Zaepfel B.L., Dickson D.W., Rigo F., Bennett F., Lusk C.P., Rothstein J.D. (2021). Nuclear accumulation of CHMP7 initiates nuclear pore complex injury and subsequent TDP-43 dysfunction in sporadic and familial ALS. Sci. Transl. Med..

[36] Farazi Fard M.A.F., Rebelo A.P., Buglo E., Nemati H., Dastsooz H., Gehweiler I., Reich S., Reichbauer J., Quintáns B., Ordóñez-Ugalde A. (2019). Truncating Mutations in UBAP1 Cause Hereditary Spastic Paraplegia. Am. J. Hum. Genet..

[37] Tomas A., Moss S.E. (2003). Calcium- and Cell Cycle-dependent Association of Annexin 11 with the Nuclear Envelope. J. Biol. Chem..

[38] Tomas A., Futter C., Moss S.E. (2004). Annexin 11 is required for midbody formation and completion of the terminal phase of cytokinesis. J. Cell Biol..

[39] Yim W.W.Y., Yamamoto H., Mizushima N. (2022). Annexins A1 and A2 are recruited to larger lysosomal injuries independently of ESCRTs to promote repair. FEBS Lett..

[40] Ebstrup M.L., Sønder S.L., Fogde D.L., Heitmann A.S.B., Dietrich T.N., Dias C., Jäättelä M., Maeda K., Nylandsted J. (2024). Annexin A7 mediates lysosome repair independently of ESCRT-III. Front. Cell Dev. Biol..

[41] Smith B.N., Topp S.D., Fallini C., Shibata H., Chen H.-J., Troakes C., King A., Ticozzi N., Kenna K.P., Soragia-Gkazi A. (2017). Mutations in the vesicular trafficking protein annexin A11 are associated with amyotrophic lateral sclerosis. Sci. Transl. Med..

[42] Couratier P., Corcia P., Lautrette G., Nicol M., Marin B. (2017). ALS and frontotemporal dementia belong to a common disease spectrum. Rev. Neurol. (Paris).

[43] Lashuel H.A., Hartley D., Petre B.M., Walz T., Lansbury P.T. (2002). Neurodegenerative disease: Amyloid pores from pathogenic mutations. Nature.

[44] Shirwany N.A., Payette D., Xie J., Guo Q. (2007). The amyloid beta ion channel hypothesis of Alzheimer’s disease. Neuropsychiatr. Dis. Treat..

[45] Viles J.H. (2023). Imaging Amyloid-β Membrane Interactions: Ion-Channel Pores and Lipid-Bilayer Permeability in Alzheimer’s Disease. Angew. Chem. Weinheim. Bergstr. Ger..

[46] Quist A., Doudevski I., Lin H., Azimova R., Ng D., Frangione B., Kagan B., Ghiso J., Lal R. (2005). Amyloid ion channels: A common structural link for protein-misfolding disease. Proc. Natl. Acad. Sci. USA.

[47] Sanderson J.B., De S., Jiang H., Rovere M., Jin M., Zaccagnini L., Hays Watson A., De Boni L., Lagomarsino V.N., Young-Pearse T.L. (2020). Analysis of α-synuclein species enriched from cerebral cortex of humans with sporadic dementia with Lewy bodies. Brain Commun..

[48] Rose K., Jepson T., Shukla S., Maya-Romero A., Kampmann M., Xu K., Hurley J.H. (2024). Tau fibrils induce nanoscale membrane damage and nucleate cytosolic tau at lysosomes. Proc. Natl. Acad. Sci. USA.

[49] Hardy J. (2017). Membrane damage is at the core of Alzheimer’s disease. Lancet Neurol..

[50] Cheeseman I.M., Desai A. (2005). A Combined Approach for the Localization and Tandem Affinity Purification of Protein Complexes from Metazoans. Sci. STKE.

[51] Gatta A.T., Olmos Y., Stoten C.L., Chen Q., Rosenthal P.B., Carlton J.G. (2021). CDK1 controls CHMP7-dependent nuclear envelope reformation. eLife.

[52] Urwin H., Authier A., Nielsen J.E., Metcalf D., Powell C., Froud K., Malcolm D.S., Holm I., Johannsen P., Brown J. (2010). Disruption of endocytic trafficking in frontotemporal dementia with CHMP2B mutations. Hum. Mol. Genet..

[53] Clayton E.L., Milioto C., Muralidharan B., Norona F.E., Edgar J.R., Soriano A., Jafar-Nejad P., Rigo F., Collinge J., Isaacs A.M. (2018). Frontotemporal dementia causative CHMP2B impairs neuronal endolysosomal traffic-rescue by TMEM106B knockdown. Brain.

[54] Thaller D.J., Allegretti M., Borah S., Ronchi P., Beck M., Lusk C.P. (2019). An ESCRT-LEM protein surveillance system is poised to directly monitor the nuclear envelope and nuclear transport system. eLife.

[55] Vietri M., Schultz S.W., Bellanger A., Jones C.M., Petersen L.I., Raiborg C., Skarpen E., Pedurupillay C.R.J., Kjos I., Kip E. (2020). Unrestrained ESCRT-III drives micronuclear catastrophe and chromosome fragmentation. Nat. Cell Biol..

[56] Olmos Y., Hodgson L., Mantell J., Verkade P., Carlton J.G. (2015). ESCRT-III controls nuclear envelope reformation. Nature.

[57] Jumper J., Evans R., Pritzel A., Green T., Figurnov M., Ronneberger O., Tunyasuvunakool K., Bates R., Žídek A., Potapenko A. (2021). Highly accurate protein structure prediction with AlphaFold. Nature.

[58] Stauffer D.R., Howard T.L., Nyun T., Hollenberg S.M. (2001). CHMP1 is a novel nuclear matrix protein affecting chromatin structure and cell-cycle progression. J. Cell Sci..

[59] Jouvenet N., Zhadina M., Bieniasz P.D., Simon S.M. (2011). Dynamics of ESCRT protein recruitment during retroviral assembly. Nat. Cell Biol..

[60] Wallen C.A., Higashikubo R., Dethlefsen L.A. (1982). Comparison of two flow cytometric assays for cellular RNA—acridine orange and propidium iodide. Cytometry.

[61] Ritter A.T., Shtengel G., Xu C.S., Weigel A., Hoffman D.P., Freeman M., Iyer N., Alivodej N., Ackerman D., Voskoboinik I. (2022). ESCRT-mediated membrane repair protects tumor-derived cells against T cell attack. Science.

[62] Satoh H., Nakano Y., Shibata H., Maki M. (2002). The penta-EF-hand domain of ALG-2 interacts with amino-terminal domains of both annexin VII and annexin XI in a Ca2+-dependent manner. Biochim. Biophys. Acta.

[63] Satoh H., Shibata H., Nakano Y., Kitaura Y., Maki M. (2002). ALG-2 Interacts with the Amino-Terminal Domain of Annexin XI in a Ca2+-Dependent Manner. Biochem. Biophys. Res. Commun..

[64] Liao Y.-C., Fernandopulle M.S., Wang G., Choi H., Hao L., Drerup C.M., Patel R., Qamar S., Nixon-Abell J., Shen Y. (2019). RNA Granules Hitchhike on Lysosomes for Long-Distance Transport, Using Annexin A11 as a Molecular Tether. Cell.

[65] Nixon-Abell J., Ruggeri F.S., Qamar S., Herling T.W., Czekalska M.A., Shen Y., Wang G., King C., Fernandopulle M.S., Sneideris T. (2025). ANXA11 biomolecular condensates facilitate protein-lipid phase coupling on lysosomal membranes. Nat. Commun..

[66] Nahm M., Lim S.M., Kim Y.-E., Park J., Noh M.-Y., Lee S., Roh J.E., Hwang S.-M., Park C.-K., Kim Y.H. (2020). ANXA11 mutations in ALS cause dysregulation of calcium homeostasis and stress granule dynamics. Sci. Transl. Med..

[67] Snyder A., Ryan V.H., Hawrot J., Lawton S., Ramos D.M., Qi Y.A., Johnson K.R., Reed X., Johnson N.L., Kollasch A.W. (2024). An ANXA11 P93S variant dysregulates TDP-43 and causes corticobasal syndrome. Alzheimers Dement..

[68] Moss S.E., Morgan R.O. (2004). The annexins. Genome Biol..

[69] Romero M., Keyel M., Shi G., Bhattacharjee P., Roth R., Heuser J.E., Keyel P.A. (2017). Intrinsic repair protects cells from pore-forming toxins by microvesicle shedding. Cell Death Differ..

[70] Keyel P.A., Loultcheva L., Roth R., Salter R.D., Watkins S.C., Yokoyama W.M., Heuser J.E. (2011). Streptolysin O clearance through sequestration into blebs that bud passively from the plasma membrane. J. Cell Sci..

[71] Gong Y.-N., Guy C., Olauson H., Becker J.U., Yang M., Fitzgerald P., Linkermann A., Green D.R. (2017). ESCRT-III Acts Downstream of MLKL to Regulate Necroptotic Cell Death and Its Consequences. Cell.

[72] Johnson B.B., Breña M., Anguita J., Heuck A.P. (2017). Mechanistic Insights into the Cholesterol-dependent Binding of Perfringolysin O-based Probes and Cell Membranes. Sci. Rep..

[73] Wade K.R., Hotze E.M., Kuiper M.J., Morton C.J., Parker M.W., Tweten R.K. (2015). An intermolecular electrostatic interaction controls the prepore-to-pore transition in a cholesterol-dependent cytolysin. Proc. Natl. Acad. Sci. USA.

[74] von Appen A., LaJoie D., Johnson I.E., Trnka M.J., Pick S.M., Burlingame A.L., Ullman K.S., Frost A. (2020). LEM2 phase separation promotes ESCRT-mediated nuclear envelope reformation. Nature.

[75] Ader N.R., Chen L., Surovtsev I.V., Chadwick W.L., Rodriguez E.C., King M.C., Lusk C.P. (2023). An ESCRT grommet cooperates with a diffusion barrier to maintain nuclear integrity. Nat. Cell Biol..

[76] Wang Y., Li S., Mokbel M., May A.I., Liang Z., Zeng Y., Wang W., Zhang H., Yu F., Sporbeck K. (2024). Biomolecular condensates mediate bending and scission of endosome membranes. Nature.

[77] von Schwedler U.K. von, Stuchell M., Müller B., Ward D.M., Chung H.-Y., Morita E., Wang H.E., Davis T., He G.-P., Cimbora D.M. (2003). The Protein Network of HIV Budding. Cell.

[78] Bodon G., Chassefeyre R., Pernet-Gallay K., Martinelli N., Effantin G., Hulsik D.L., Belly A., Goldberg Y., Chatellard-Causse C., Blot B. (2011). Charged Multivesicular Body Protein 2B (CHMP2B) of the Endosomal Sorting Complex Required for Transport-III (ESCRT-III) Polymerizes into Helical Structures Deforming the Plasma Membrane. J. Biol. Chem..

[79] Franceschi N.D., Alqabandi M., Miguet N., Caillat C., Mangenot S., Weissenhorn W., Bassereau P. (2018). The ESCRT protein CHMP2B acts as a diffusion barrier on reconstituted membrane necks. J. Cell Sci..

[80] Clayton E.L., Mizielinska S., Edgar J.R., Nielsen T.T., Marshall S., Norona F.E., Robbins M., Damirji H., Holm I.E., Johannsen P. (2015). Frontotemporal dementia caused by CHMP2B mutation is characterised by neuronal lysosomal storage pathology. Acta Neuropathol..

[81] Jun Y.-W., Hass E.P., Lee S., Fazzio T.G., Gao F.-B. (2026). Mislocalization of FTD3-associated mutant CHMP2B to the nucleus of human neurons due to loss of a nuclear export signal. Acta Neuropathol. Commun..

[82] Ghazi-Noori S., Froud K.E., Mizielinska S., Powell C., Smidak M., Fernandez de Marco M.F. de, O’Malley C., Farmer M., Parkinson N., Fisher E.M.C. (2012). Progressive neuronal inclusion formation and axonal degeneration in CHMP2B mutant transgenic mice. Brain.

[83] Carlton J.G., Martin-Serrano J. (2007). Parallels Between Cytokinesis and Retroviral Budding: A Role for the ESCRT Machinery. Science.

[84] Rueden C.T., Schindelin J., Hiner M.C., DeZonia B.E., Walter A.E., Arena E.T., Eliceiri K.W. (2017). ImageJ2: ImageJ for the next generation of scientific image data. BMC Bioinform..

[85] Hennies J., Lleti J.M.S., Schieber N.L., Templin R.M., Steyer A.M., Schwab Y. (2020). AMST: Alignment to Median Smoothed Template for Focused Ion Beam Scanning Electron Microscopy Image Stacks. Sci. Rep..

[86] Bogovic, J.A., Hanslovsky, P., Wong, A., and Saalfeld, S. (2016). Robust registration of calcium images by learned contrast synthesis. 2016 IEEE 13th International Symposium on Biomedical Imaging (ISBI) (IEEE) pp. 1123–1126. 10.1109/ISBI.2016.7493463.

[87] Cardona A., Saalfeld S., Schindelin J., Arganda-Carreras I., Preibisch S., Longair M., Tomancak P., Hartenstein V., Douglas R.J. (2012). TrakEM2 software for neural circuit reconstruction. PLoS One.

[88] Pearson G.J., Mears H.V., Broncel M., Snijders A.P., Bauer D.L.V., Carlton J.G. (2024). ER-export and ARFRP1/AP-1–dependent delivery of SARS-CoV-2 Envelope to lysosomes controls late stages of viral replication. Sci. Adv..

[89] Mierzwa B.E., Chiaruttini N., Redondo-Morata L., von Filseck J.M. von, König J., Larios J., Poser I., Müller-Reichert T., Scheuring S., Roux A. (2017). Dynamic subunit turnover in ESCRT-III assemblies is regulated by Vps4 to mediate membrane remodelling during cytokinesis. Nat. Cell Biol..

[90] Shamonin D.P., Bron E.E., Lelieveldt B.P.F., Smits M., Klein S., Staring M., Alzheimer's Disease Neuroimaging Initiative (2014). Fast parallel image registration on CPU and GPU for diagnostic classification of Alzheimer’s disease. Front. Neuroinform..

[91] Marstal, K., Berendsen, F., Staring, M., and Klein, S. (2016). SimpleElastix: A User-Friendly, Multi-lingual Library for Medical Image Registration. 2016 IEEE Conference on Computer Vision and Pattern Recognition Workshops (CVPRW) (IEEE) pp. 574–582. 10.1109/CVPRW.2016.78.

[92] Ntatsis, K., Dekker, N., van der Valk, V., Birdsong, T., Zukić, D., Klein, S., Staring, M., and McCormick, M. (2023). itk-elastix: Medical image registration in Python. Proceedings of the Python in Science Conference, 101–105. 10.25080/gerudo-f2bc6f59-00d.

