# Supplementary material for: The ALS- and FTD-associated proteins annexin A11 and CHMP2B act sequentially in plasma membrane repair
Source: Dev Cell. 2026 Jul 8;61(7):1487–1503.e7. doi: 10.1016/j.devcel.2026.05.014 (PMC13374510; doi:10.1016/j.devcel.2026.05.014)
Supplement: Document S1. Figures S1–S7 [file mmc1.pdf]

Developmental Cell, Volume *61*

Supplemental information

The ALS- and FTD-associated proteins annexin A11 and CHMP2B act sequentially in plasma membrane repair

Catherine M. Heffner, Georgina P. Starling, Lorian C. Straker, Philippa C. Hawes, Adrian M. Isaacs, and Jeremy G. Carlton

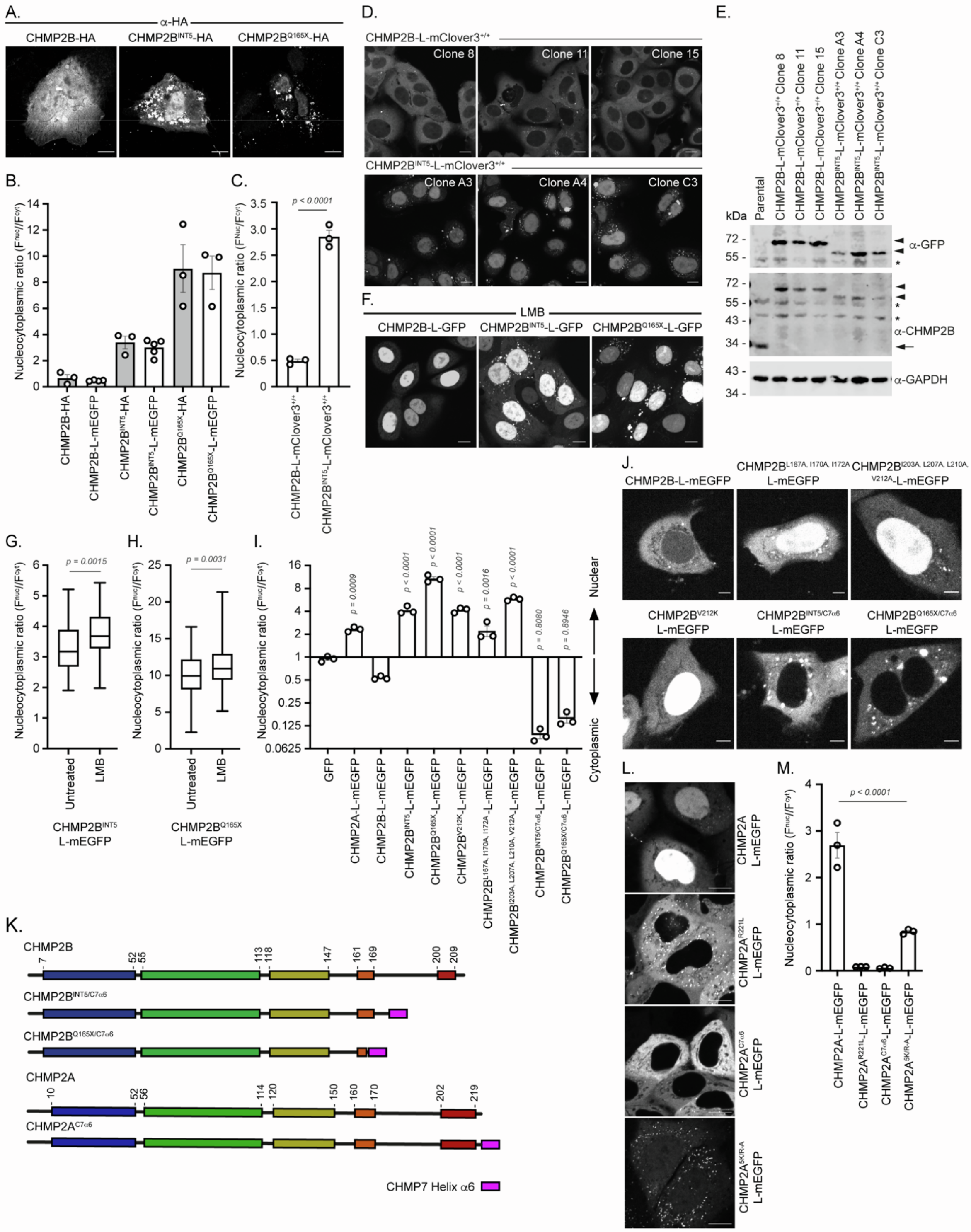

A.
α-HA
CHMP2B-HA
CHMP2B^{INT5}-HA
CHMP2B^{Q165X}-HA
B.
Nucleocytoplasmic ratio (F^{nuc}/F^{cyt})
CHMP2B-HA
CHMP2B-L-mEGFP
CHMP2B^{INT5}-HA
CHMP2B^{INT5}-L-mEGFP
CHMP2B^{Q165X}-HA
CHMP2B^{Q165X}-L-mEGFP
C.
p < 0.0001
CHMP2B-L-mClover3$^{+/+}$
CHMP2B^{INT5}-L-mClover3$^{+/+}$
D.
CHMP2B-L-mClover3$^{+/+}$
Clone 8
Clone 11
Clone 15
CHMP2B^{INT5}-L-mClover3$^{+/+}$
Clone A3
Clone A4
Clone C3
E.
kDa
Parental
CHMP2B-L-mClover3$^{+/+}$ Clone 8
CHMP2B-L-mClover3$^{+/+}$ Clone 11
CHMP2B-L-mClover3$^{+/+}$ Clone 15
CHMP2B^{INT5}-L-mClover3$^{+/+}$ Clone A3
CHMP2B^{INT5}-L-mClover3$^{+/+}$ Clone A4
CHMP2B^{INT5}-L-mClover3$^{+/+}$ Clone C3
α-GFP
α-CHMP2B
α-GAPDH
F.
LMB
CHMP2B-L-GFP
CHMP2B^{INT5}-L-GFP
CHMP2B^{Q165X}-L-GFP
G.
p = 0.0015
Untreated
LMB
CHMP2B^{INT5} L-mEGFP
H.
p = 0.0031
Untreated
LMB
CHMP2B^{Q165X} L-mEGFP
I.
Nuclear
Cytoplasmic
p = 0.0009
p < 0.0001
p < 0.0001
p < 0.0001
p = 0.0016
p < 0.0001
p = 0.8080
p = 0.8946
GFP
CHMP2A-L-mEGFP
CHMP2B-L-mEGFP
CHMP2B^{INT5}-L-mEGFP
CHMP2B^{Q165X}-L-mEGFP
CHMP2B^{V212K}-L-mEGFP
CHMP2B$^{L167A, I170A, I172A}$-L-mEGFP
CHMP2B$^{I203A, L207A, L210A, V212A}$-L-mEGFP
CHMP2B$^{INT5/C7α6}$-L-mEGFP
CHMP2B$^{Q165X/C7α6}$-L-mEGFP
J.
CHMP2B-L-mEGFP
CHMP2B$^{L167A, I170A, I172A}$ L-mEGFP
CHMP2B$^{I203A, L207A, L210A, V212A}$-L-mEGFP
CHMP2B^{V212K} L-mEGFP
CHMP2B$^{INT5/C7α6}$ L-mEGFP
CHMP2B$^{Q165X/C7α6}$ L-mEGFP
K.
CHMP2B
CHMP2B$^{INT5/C7α6}$
CHMP2B$^{Q165X/C7α6}$
CHMP2A
CHMP2A^{C7α6}
CHMP7 Helix α6
L.
CHMP2A L-mEGFP
CHMP2A^{R221L} L-mEGFP
CHMP2A^{C7α6} L-mEGFP
CHMP2A$^{5K/R-A}$ L-mEGFP
M.
p < 0.0001
CHMP2A-L-mEGFP
CHMP2A^{R221L}-L-mEGFP
CHMP2A^{C7α6}-L-mEGFP
CHMP2A$^{5K/R-A}$-L-mEGFP

Figure S1. CHMP2B encodes C-terminal nuclear export sequences that are truncated by pathogenic FTD-causing mutations, related to Figure 1.

(A) Representative images of CAL-51 cells transiently transfected with HA-tagged CHMP2B and illuminated with anti-HA antibodies. Scale bars represent 10 μm.

(B) Quantification of nucleocytoplasmic ratio of HA-tag fluorescence from cells in A or compared to the CAL-51 cell lines stably expressing equivalent CHMP2B-L-mEGFP fusion proteins (GFP data reused from Fig.1B). CHMP2B-HA (n = 26, N = 3), CHMP2B^{INT5}-HA (n = 30, N =3), CHMP2B^{Q165X}-HA (n = 26, N = 3). CHMP2B-L-mEGFP (n = 57, N = 4), CHMP2B^{INT5}-L-mEGFP (n = 31, N = 5), CHMP2B^{Q165X}-L-mEGFP (n = 104, N = 3). Graphs show mean ± S.E.M. with significance between HA and mEGFP equivalent cells calculated by two-tailed T-test (all non-significant).

(C-E) Nucleocytoplasmic quantification (C), representative images (D) and western blotting (E) of 3 independent clones of CAL-51 cells homozygously edited to express CHMP2B-L-mClover3$^{+/+}$ or CHMP2B^{INT5}-L-mClover3$^{+/+}$. In images, Scale bar is 10 μm. Quantification of nucleocytoplasmic ratio from the indicated number of cells. CHMP2B-L-mClover3$^{+/+}$ Clone 8, n = 96; Clone 11, n = 69; Clone 15, n = 112. CHMP2B^{INT5}-L-mClover3$^{+/+}$ Clone A3, n = 49; Clone A4, n = 85; Clone C3, n = 61. Graph displays mean ± S.E.M. from the N = 3 independent clones for each edit. Significance between conditions was calculated by 2-tailed T-test. In western blot, resolved lysates of the indicated parental or edited CAL-51 cell lines were examined by western blotting with antibodies that detect GFP, CHMP2B or GAPDH. Endogenous CHMP2B indicated by arrowhead; edited CHMP2B-L-mClover3$^{+/+}$ or CHMP2B^{INT5}-L-mClover3$^{+/+}$ indicated by arrows. Non-specific bands indicated by asterisks.

(F-H) Representative images (F) and quantification (G-H) of nucleocytoplasmic partitioning in CAL-51 cells stably expressing CHMP2B-L-mEGFP, CHMP2B^{INT5}-L-mEGFP or CHMP2B^{Q165X}-L-mEGFP and treated with LMB (10 ng/mL, 4 hours). In F, scale bars represent 10 μm. Quantification (G and H) of nucleocytoplasmic partitioning of cells expressing CHMP2B^{INT5}-L-mEGFP and treated with LMB (n = 36 (untreated) or 73 (+LMB)) or CHMP2B-L-mEGFP^{Q165X} (n = 72 (untreated) or 85 (+LMB)); related to Figure 1D. Data displayed as a box and whisker plot with median, 25th and 75th percentile displayed. Significance calculated by 2-tailed T-test.

(I, J) Quantification and representative live images of CAL-51 cells transiently transfected with mEGFP (n = 18), CHMP2A-L-mEGFP (n = 50), CHMP2B-L-mEGFP, (n = 41), CHMP2B^{INT5}-L-mEGFP (n = 37), CHMP2B^{Q165X}-L-mEGFP (n = 40), CHMP2B$^{L167A/I170A/I172A}$-L-mEGFP, (n = 46), CHMP2B$^{I203A/L207A/L210A/V212A}$-L-mEGFP (n = 56), CHMP2B^{V212K}-L-mEGFP, (n = 67), CHMP2B$^{INT5/C7\alpha6}$-L-mEGFP, (n = 43), CHMP2B$^{Q165X/C7\alpha6}$-L-mEGFP (n = 54), graph in I displays mean ± S.E.M. from N = 3 independent experiments with significance calculated by one-way ANOVA. P values are depicted from multiple comparisons relative to CHMP2B-L-GFP. Related to Figure 1C. Scale bars in J represent 5 μm.

(K) Schematic depicting chimaeric sequences of CHMP2A, CHMP2B^{INT5} and CHMP2B^{Q165X} fused with sequences from CHMP7 Helix α6 containing a type-1 NES. Helix boundaries depicted by alternating colours and placed according to Alphafold-2 predictions of CHMP2B (Q9UQN3). Addition of CHMP7's NES sequence indicated by magenta box.

(L, M) Representative images and quantification of the nucleocytoplasmic ratio of the GFP signal in cells stably expressing CHMP2A-L-mEGFP (n = 69), CHMP2A^{R221L}-L-mEGFP (n = 116), CHMP2A$^{C7\alpha6}$-L-mEGFP (n = 97) or CHMP2A$^{5K/R-A}$-L-mEGFP (n = 85). Graph displays mean ± S.E.M from N = 3 independent experiments, significance calculated by one-way ANOVA. In L scale bar is 10 μm.

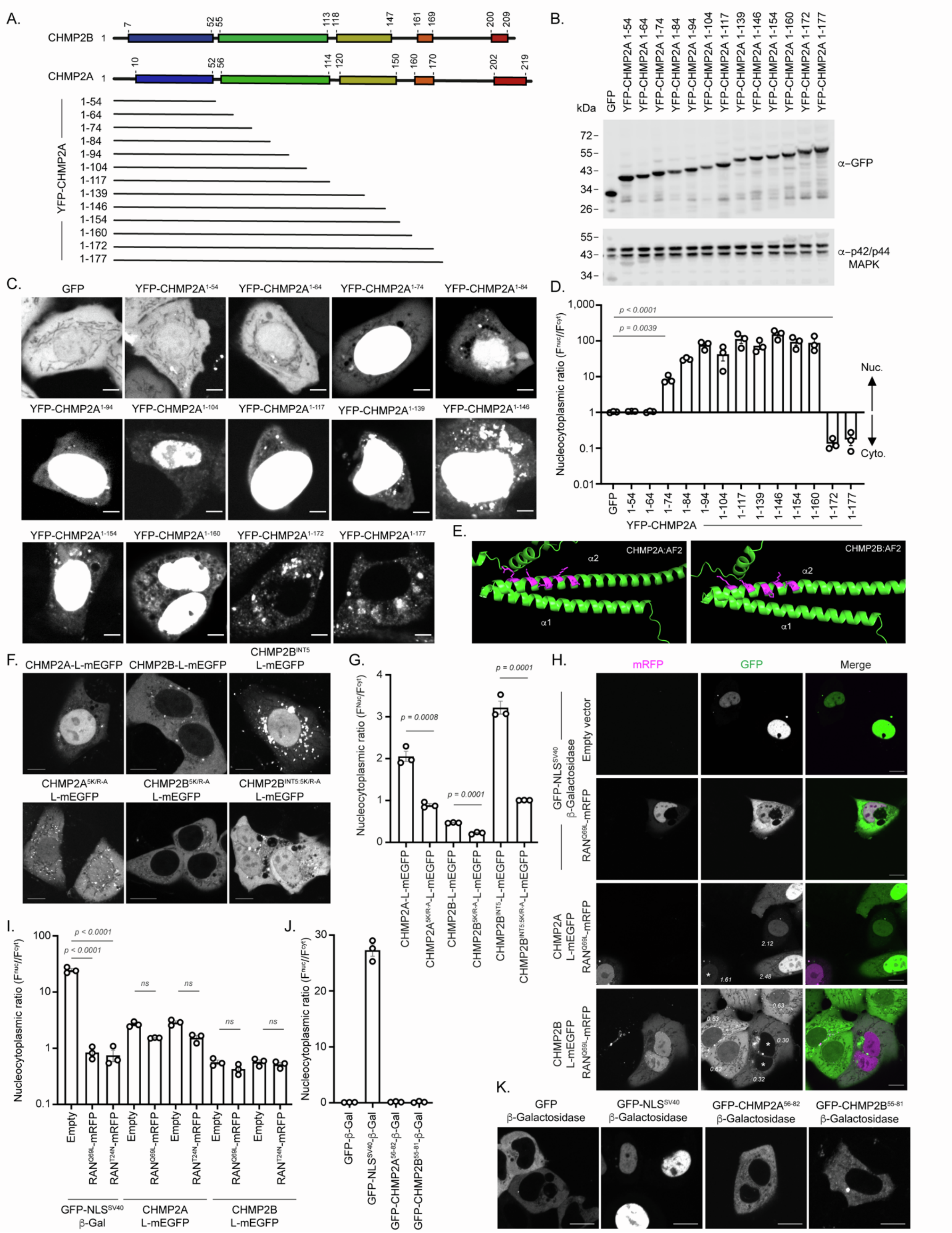
A.
CHMP2B
CHMP2A
YFP-CHMP2A
1-54
1-64
1-74
1-84
1-94
1-104
1-117
1-139
1-146
1-154
1-160
1-172
1-177
B.
kDa
GFP
YFP-CHMP2A 1-54
YFP-CHMP2A 1-64
YFP-CHMP2A 1-74
YFP-CHMP2A 1-84
YFP-CHMP2A 1-94
YFP-CHMP2A 1-104
YFP-CHMP2A 1-117
YFP-CHMP2A 1-139
YFP-CHMP2A 1-146
YFP-CHMP2A 1-154
YFP-CHMP2A 1-160
YFP-CHMP2A 1-172
YFP-CHMP2A 1-177
α-GFP
α-p42/p44 MAPK
C.
GFP
YFP-CHMP2A1-54
YFP-CHMP2A1-64
YFP-CHMP2A1-74
YFP-CHMP2A1-84
YFP-CHMP2A1-94
YFP-CHMP2A1-104
YFP-CHMP2A1-117
YFP-CHMP2A1-139
YFP-CHMP2A1-146
YFP-CHMP2A1-154
YFP-CHMP2A1-160
YFP-CHMP2A1-172
YFP-CHMP2A1-177
D.
p < 0.0001
p = 0.0039
Nucleocytoplasmic ratio (Fnuc/Fcyt)
Nuc.
Cyto.
YFP-CHMP2A
E.
CHMP2A:AF2
CHMP2B:AF2
α1
α2
F.
CHMP2A-L-mEGFP
CHMP2B-L-mEGFP
CHMP2BINT5 L-mEGFP
CHMP2A5K/R-A L-mEGFP
CHMP2B5K/R-A L-mEGFP
CHMP2BINT5:5K/R-A L-mEGFP
G.
p = 0.0008
p = 0.0001
p = 0.0001
Nucleocytoplasmic ratio (Fnuc/Fcyt)
CHMP2A-L-mEGFP
CHMP2A5K/R-A-L-mEGFP
CHMP2B-L-mEGFP
CHMP2B5K/R-A-L-mEGFP
CHMP2BINT5-L-mEGFP
CHMP2BINT5:5K/R-A-L-mEGFP
H.
mRFP
GFP
Merge
GFP-NLSSV40 β-Galactosidase
Empty vector
RANQ69L-mRFP
CHMP2A L-mEGFP RANQ69L-mRFP
CHMP2B L-mEGFP RANQ69L-mRFP
I.
p < 0.0001
p < 0.0001
ns
Nucleocytoplasmic ratio (Fnuc/Fcyt)
Empty
RANQ69L-mRFP
RANT24N-mRFP
GFP-NLSSV40 β-Gal
CHMP2A L-mEGFP
CHMP2B L-mEGFP
J.
Nucleocytoplasmic ratio (Fnuc/Fcyt)
GFP-β-Gal
GFP-NLSSV40-β-Gal
GFP-CHMP2A56-82-β-Gal
GFP-CHMP2B55-81-β-Gal
K.
GFP β-Galactosidase
GFP-NLSSV40 β-Galactosidase
GFP-CHMP2A56-82 β-Galactosidase
GFP-CHMP2B55-81 β-Galactosidase

Figure S2. Identification of nuclear retention sequences in the α2 helix of CHMP2 proteins, related to Figure 1.

(A) Schematic representation of YFP-CHMP2A deletions (described in[56]).

(B) Resolved lysates from 293T cells transfected with vectors encoding mEGFP, or the YFP-CHMP2A deletions from A were examined by western blotting with antibodies that detect EGFP or p42/p44 MAPK.

(C) Representative images of CAL-51 cells transiently transfected with mEGFP or the constructs depicted in (A) and imaged live. Scale bars represent 5 μm.

(D) Quantification of nucleocytoplasmic ratio of YFP fluorescence from CAL-51 cells transfected with mEGFP (n = 81) YFP-CHMP2A$^{1\text{-}54}$ (n = 53), YFP-CHMP2A$^{1\text{-}64}$ (n = 66), YFP-CHMP2A$^{1\text{-}74}$ (n = 49), YFP-CHMP2A$^{1\text{-}84}$ (n = 36), YFP-CHMP2A$^{1\text{-}94}$ (n = 47), YFP-CHMP2A$^{1\text{-}104}$ (n = 50), YFP-CHMP2A$^{1\text{-}117}$ (n = 49), YFP-CHMP2A$^{1\text{-}139}$ (n = 45), YFP-CHMP2A$^{1\text{-}146}$ (n = 44), YFP-CHMP2A$^{1\text{-}154}$ (n = 34), YFP-CHMP2A$^{1\text{-}160}$ (n = 81), YFP-CHMP2A$^{1\text{-}172}$ (n = 50), YFP-CHMP2A$^{1\text{-}177}$ (n = 47). Graphs show mean ± S.E.M. from N = 3 independent experiments with significance between select conditions calculated by two tailed T-test.

(E) Alphafold-2 models of CHMP2A (O43633) or CHMP2B (Q9UQN3) displaying the α1 - α2 hairpin with basic residues on the surface of α2 highlighted in magenta.

(F) Representative images of CAL-51 cells expressing the indicated CHMP2 proteins. CHMP2A$^{5K/R\text{-}A}$-L-mEGFP contains mutations R60A/K64A/R68A/R71A/K75A. CHMP2B$^{5K/R\text{-}A}$-L-mEGFP and CHMP2B$^{INT5:5K/R\text{-}A}$-L-mEGFP contain mutations K59A/K63A/H67A/K70A/R74A. Scale bar is 10 μm.

(G) Quantification of nucleocytoplasmic ratio from cells in F. CHMP2A-L-mEGFP (n = 42), CHMP2A$^{5K/R\text{-}A}$-L-mEGFP (n = 52), CHMP2B-L-mEGFP (n = 35), CHMP2B$^{5K/R\text{-}A}$-L-mEGFP (n = 48), CHMP2B^{INT5}-L-mEGFP (n = 43) or CHMP2B$^{INT5:5K/R\text{-}A}$-L-mEGFP (n = 56). Graph displays mean ± S.E.M. from N = 3 independent experiments. Significance calculated by 2-tailed T-test between conditions.

(H, I) Representative images (H) and nucleocytoplasmic ratio (I) of GFP-signals in CAL-51 cells co-transfected with GFP-NLS^{SV40}-β-Galactosidase, CHMP2A-L-mEGFP or CHMP2B-L-mEGFP and either empty vector, or vectors encoding RAN^{Q69L}-mRFP1 or RAN^{T24N}-mRFP1. Graph displays mean ± S.E.M. from N = 3 independent experiments with 20-73 transfected cells acquired per condition. Significance calculated by one-way ANOVA. In H, nucleocytoplasmic ratio of CHMP2A-L-mEGFP or CHMP2B-L-mEGFP overlayed on cells transfected or not with RAN^{Q69L}-mRFP1 (asterisked). In H, scale bar is 10 μm.

(J, K) Quantification (J) and representative images (K) of CAL-51 cells transfected with plasmids encoding GFP-β-Galactosidase (n = 69), GFP-NLS^{SV40}-β-Galactosidase (n = 46), GFP-CHMP2A$^{56\text{-}82}$-β-Galactosidase (n = 33) or GFP-CHMP2B$^{55\text{-}81}$-β-Galactosidase (n = 17). Graph displays mean ± S.E.M. from N = 3 independent experiments. In K, scale bar is 10 μm.

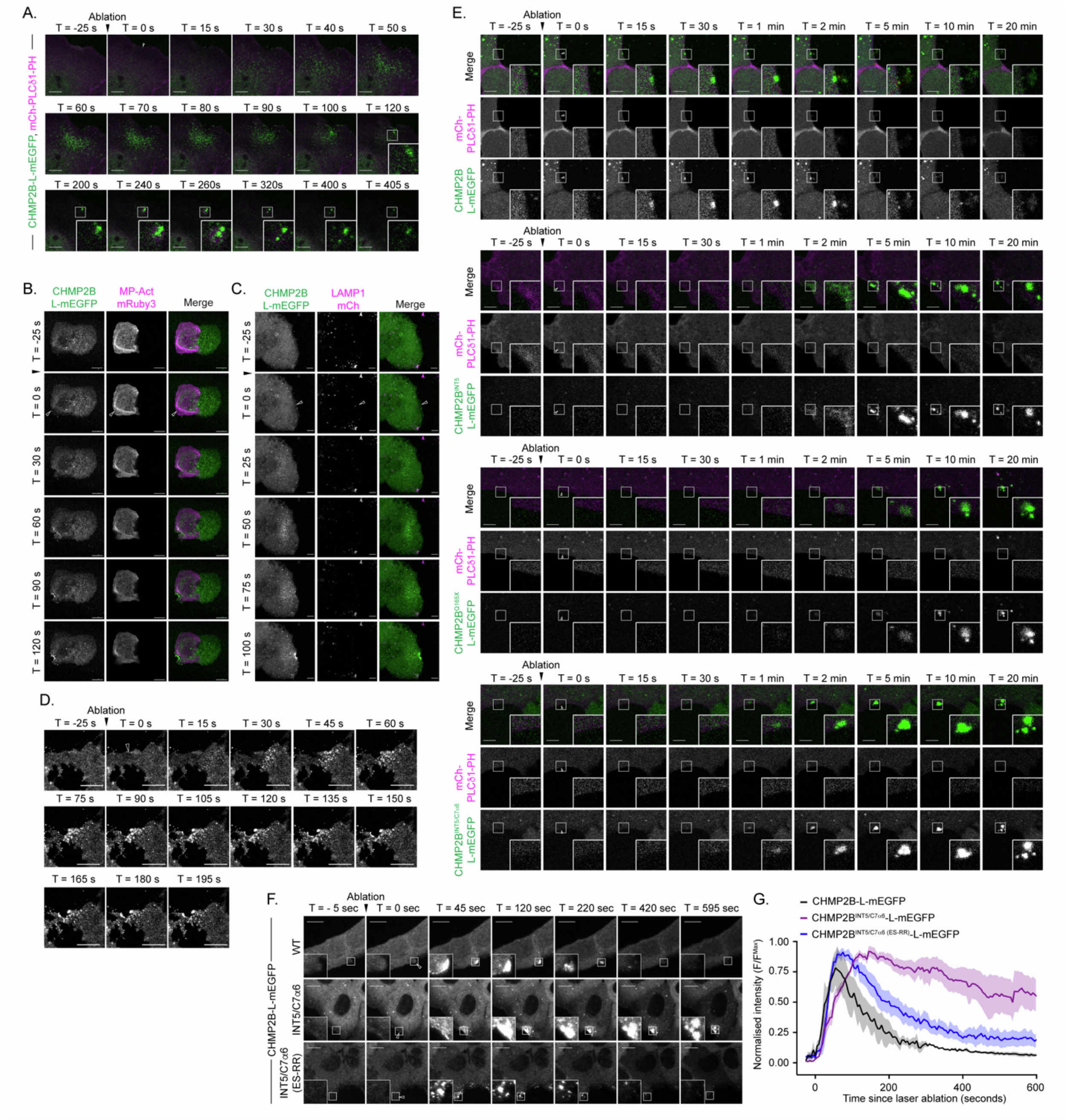

A.
Ablation
T = -25 s
T = 0 s
T = 15 s
T = 30 s
T = 40 s
T = 50 s
T = 60 s
T = 70 s
T = 80 s
T = 90 s
T = 100 s
T = 120 s
T = 200 s
T = 240 s
T = 260s
T = 320s
T = 400 s
T = 405 s
CHMP2B-L-mEGFP, mCh-PLCδ1-PH
B.
CHMP2B L-mEGFP
MP-Act mRuby3
Merge
T = -25 s
T = 0 s
T = 30 s
T = 60 s
T = 90 s
T = 120 s
C.
CHMP2B L-mEGFP
LAMP1 mCh
Merge
T = -25 s
T = 0 s
T = 25 s
T = 50 s
T = 75 s
T = 100 s
D.
Ablation
T = -25 s
T = 0 s
T = 15 s
T = 30 s
T = 45 s
T = 60 s
T = 75 s
T = 90 s
T = 105 s
T = 120 s
T = 135 s
T = 150 s
T = 165 s
T = 180 s
T = 195 s
E.
Ablation
T = -25 s
T = 0 s
T = 15 s
T = 30 s
T = 1 min
T = 2 min
T = 5 min
T = 10 min
T = 20 min
Merge
mCh-PLCδ1-PH
CHMP2B L-mEGFP
CHMP2B^INT5 L-mEGFP
CHMP2B^Q165X L-mEGFP
CHMP2B^INT5/C7α6 L-mEGFP
F.
Ablation
T = - 5 sec
T = 0 sec
T = 45 sec
T = 120 sec
T = 220 sec
T = 420 sec
T = 595 sec
WT
INT5/C7α6
INT5/C7α6 (ES-RR)
CHMP2B-L-mEGFP
G.
CHMP2B-L-mEGFP
CHMP2B^INT5/C7α6-L-mEGFP
CHMP2B^INT5/C7α6 (ES-RR)-L-mEGFP
Normalised intensity (F/F^Max)
1.00
0.75
0.50
0.25
0.00
0
200
400
600
Time since laser ablation (seconds)

Figure S3. Characterisation of CHMP2B-L-mEGFP recruitment to sites of plasma membrane damage, related to Figure 1.

(A) Representative images of CAL-51 cells stably expressing CHMP2B-L-mEGFP, transiently transfected with a vector encoding mCh-PLCδ1-PH to illuminate the plasma membrane. Here, the site of laser ablation was placed at the edge of a lamellipodium, enabling the visualisation of CHMP2B-L-mEGFP recruitment in a stellate wave-form pattern focusing on the site of damage. Arrowhead indicates site of ablation. Scale bar is 5 µm. See corresponding Mendeley Data Video 1.

(B) Representative image sequence of CAL-51 cells stably expressing CHMP2B-L-mEGFP and transiently transfected with MP-Act-mRuby3 to illuminate membrane proximal F-actin and subject to a single multi-photon laser ablation at the plasma membrane. Scale bars represent 10 µm. Arrowhead indicates site of ablation. Images representative of n = 8. See corresponding Mendeley Data Video 2.

(C) Representative image sequence from CAL-51 cells stably expressing CHMP2B-L-mEGFP, transiently transfected with a plasmid encoding LAMP1-tdTomato and subject to a single multi-photon laser ablation at the plasma membrane. Images representative of n = 8. Scale bars represent 5 µm. Arrowhead indicates site of ablation. See corresponding Mendeley Data Video 3.

(D) Representative image sequence from CAL-51 cells stably expressing CHMP2B-L-mEGFP, pretreated with cytochalasin D (2 µg/mL, 30 minutes prior) and subject to a single multi-photon laser ablation at the plasma membrane. Scale bar is 10 µm. Arrowhead indicates site of ablation. Images representative of n = 7. See corresponding Mendeley Data Video 4.

(E) Channel separated sequence of representative images from cells described in Fig. 1G. CAL-51 cells stably expressing CHMP2B-L-mEGFP, CHMP2B^{INT5}-L-mEGFP, CHMP2B^{Q165X}-L-mEGFP or CHMP2B-L-mEGFP, CHMP2B$^{INT5/C7\alpha6}$-L-mEGFP were subject to a single multi-photon laser ablation at the mCh-PLCδ1-PH illuminated plasma membrane. Arrowheads indicate site of laser ablation. Z-plane is set below the nucleus to visualise events at the peripheral plasma membrane. Scale bar is 5 µm. See corresponding Video S1.

(F) Representative image sequences and quantification of CAL-51 cells stably expressing CHMP2B-L-mEGFP, CHMP2B$^{INT5/C7\alpha6}$-L-mEGFP or CHMP2B$^{INT5/C7\alpha6(ES\text{-}RR)}$-L-mEGFP and subject to laser ablation at the plasma membrane and imaged live. Arrowhead indicates site of ablation. Scale bar is 10 µm. See corresponding Mendeley Data Videos 5 and 6.

(G) Graph displays mean ± S.E.M. of GFP recruitment signal at site of ablation from Figure S3F. CHMP2B-L-mEGFP (n = 9, N = 4) CHMP2B$^{INT5/C7\alpha6}$-L-mEGFP (n = 12, N = 4) or CHMP2B$^{INT5/C7\alpha6(ES\text{-}RR)}$-L-mEGFP (n = 12, N = 4).

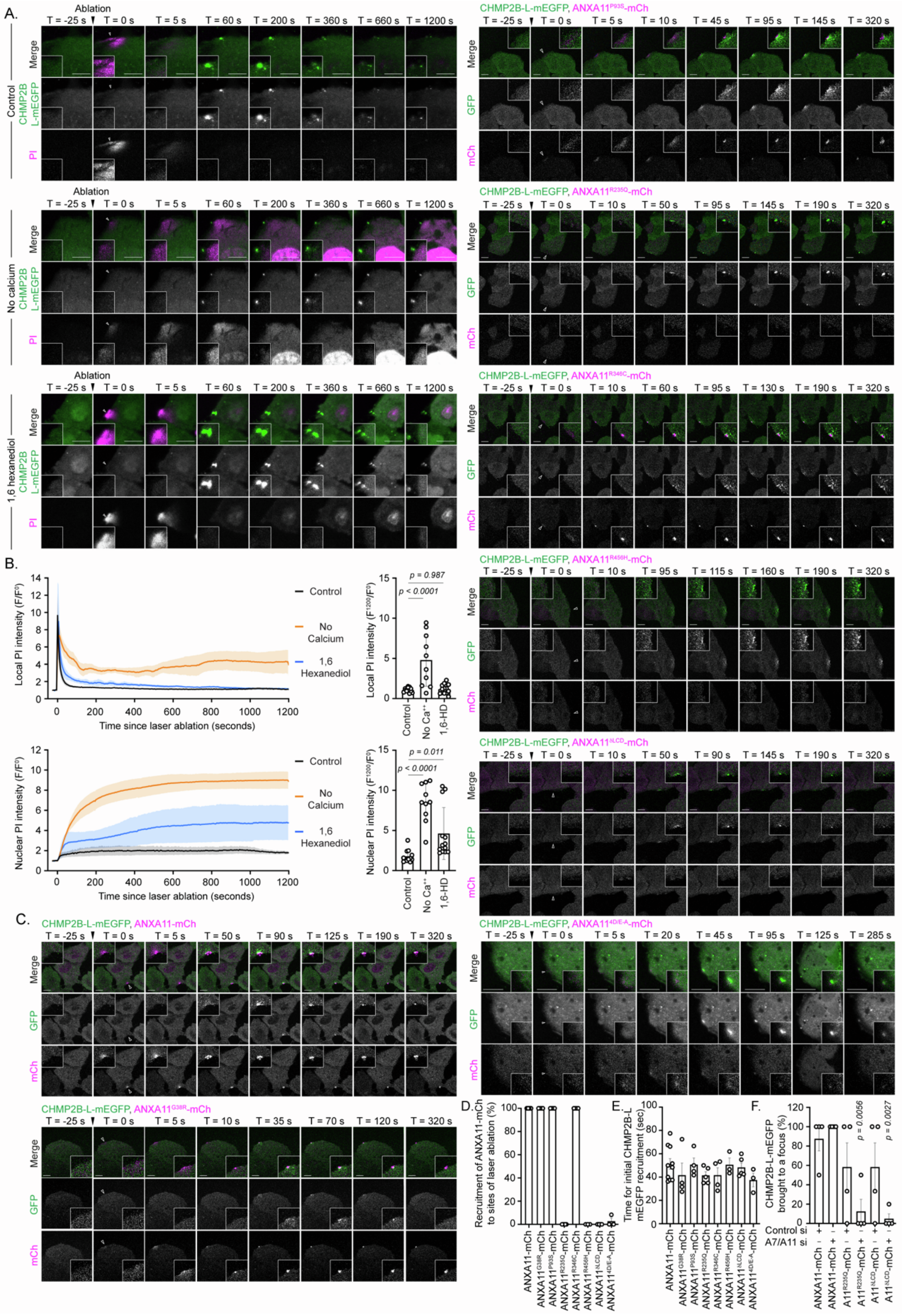
A.
Ablation
T = -25 s
T = 0 s
T = 5 s
T = 60 s
T = 200 s
T = 360 s
T = 660 s
T = 1200 s
Merge
Control
CHMP2B L-mEGFP
PI
No calcium
1,6 hexanediol
CHMP2B-L-mEGFP, ANXA11^P93S-mCh
CHMP2B-L-mEGFP, ANXA11^R235Q-mCh
CHMP2B-L-mEGFP, ANXA11^R346C-mCh
CHMP2B-L-mEGFP, ANXA11^R456H-mCh
CHMP2B-L-mEGFP, ANXA11^ΔLCD-mCh
CHMP2B-L-mEGFP, ANXA11^4D/E-A-mCh
GFP
mCh
B.
Local PI intensity (F/F⁰)
Time since laser ablation (seconds)
Control
No Calcium
1,6 Hexanediol
Local PI intensity (F¹²⁰⁰/F⁰)
p = 0.987
p < 0.0001
Control
No Ca⁺⁺
1,6-HD
Nuclear PI intensity (F/F⁰)
Nuclear PI intensity (F¹²⁰⁰/F⁰)
p = 0.011
p < 0.0001
C.
CHMP2B-L-mEGFP, ANXA11-mCh
CHMP2B-L-mEGFP, ANXA11^G38R-mCh
D.
Recruitment of ANXA11-mCh to sites of laser ablation (%)
ANXA11-mCh
ANXA11^G38R-mCh
ANXA11^P93S-mCh
ANXA11^R235Q-mCh
ANXA11^R346C-mCh
ANXA11^R456H-mCh
ANXA11^ΔLCD-mCh
ANXA11^4D/E-A-mCh
E.
Time for initial CHMP2B-L-mEGFP recruitment (sec)
F.
CHMP2B-L-mEGFP brought to a focus (%)
p = 0.0056
p = 0.0027
Control si
A7/A11 si
A11^R235Q-mCh
A11^ΔLCD-mCh

Figure S4. Activities in ANXA11 necessary for plasma membrane repair, related to Figure 3.

(A) Representative image sequences from CAL-51 cells stably expressing CHMP2B-L-mEGFP, incubated in the presence of PI (160 μg/mL) and subject to live cell imaging and laser ablation at the plasma membrane. The imaging media was either complete (top panels), calcium free (middle panels) or contained 1.25% 1,6-hexanediol. Arrowhead indicates the site of ablation. Scale bar is 10 μm. See corresponding Mendeley Data Videos 15 - 17.

(B) Quantification of PI influx from cells in A, either locally at the site of damage, or in the nucleus. Fluorescence intensities were background corrected and relative to the average of the pre-ablation frames in each case. Traces depict mean ± S.E.M (Control n = 13, N = 3; No Calcium, n = 10, N = 3; 1,6-hexanediol, n = 13, N = 3). The bar graphs depict the local or nuclear PI intensity at 20 minutes after ablation and display mean ± S.D. of all data points. Significance across individual datapoints was calculated by one-way ANOVA.

(C) Representative images of CAL-51 cells stably expressing CHMP2B-L-mEGFP and either ANXA11-mCh, ANXA11^{G38R}-mCh, ANXA11^{P93S}-mCh, ANXA11^{R235Q}-mCh, ANXA11^{R456H}-mCh or ANXA11$^{\delta LCD}$-mCh or ANXA11$^{E257A/D329A/E413A/D488A}$-mCh (ANXA11$^{4D/E\text{-}A}$-mCh) and subject to a single multi-photon laser ablation at the plasma membrane. Recruitment of CHMP2B-L-mEGFP (enlarged in boxed region) was used as a positive indicator of membrane damage. In microscopy images, scale bar is 10 μm. See corresponding Mendeley Data Videos 18 - 25.

(D) Frequency of wildtype and mutant ANXA11-mCh recruitment to sites of laser-ablation from C. Graph displays mean ± S.E.M. CHMP2B-L-mEGFP:ANXA11-mCh (n = 29, N = 10); CHMP2B-L-mEGFP:ANXA11^{G38R}-mCh (n = 8, N = 4); CHMP2B-L-mEGFP:ANXA11^{P93S}-mCh (n = 11, N = 4); CHMP2B-L-mEGFP:ANXA11^{R235Q}-mCh (n = 11, N = 5); CHMP2B-L-mEGFP:ANXA11^{R346C}-mCh (n = 12, N = 4); CHMP2B-L-mEGFP:ANXA11^{R456H}-mCh (n = 11, N = 3); CHMP2B-L-mEGFP:ANXA11$^{\delta LCD}$-mCh (n = 16, N = 6), CHMP2B-L-mEGFP:ANXA11$^{4D/E\text{-}A}$-mCh (n = 18, N = 3).

(E) Quantification of CHMP2B-L-mEGFP recruitment time in cells expressing the indicated ANXA11-mCh proteins. Graph displays mean ± S.E.M. from the indicated number of independent experiments. CHMP2B-L-mEGFP:ANXA11-mCh (n = 29, N = 10); CHMP2B-L-mEGFP:ANXA11^{G38R}-mCh (n = 8, N = 4); CHMP2B-L-mEGFP:ANXA11^{P93S}-mCh (n = 11, N = 4); CHMP2B-L-mEGFP:ANXA11^{R235Q}-mCh (n = 11, N = 5); CHMP2B-L-mEGFP:ANXA11^{R346C}-mCh (n = 12, N = 4); CHMP2B-L-mEGFP:ANXA11^{R456H}-mCh (n = 11, N = 3); CHMP2B-L-mEGFP:ANXA11$^{\delta LCD}$-mCh (n = 14, N = 5), CHMP2B-L-mEGFP:ANXA11$^{4D/E\text{-}A}$-mCh (n = 18, N = 3). No significant differences in CHMP2B-L-mEGFP recruitment time, calculated by one-way ANOVA, were observed across these cell lines.

(F) Quantification of whether the broad CHMP2B-L-mEGFP recruitment was brought to a focus at the site of membrane damage. Graphs show mean ± S.E.M. CHMP2B-L-mEGFP:ANXA11-mCh (control siRNA, n = 9, N = 4; ANXA7/ANXA11 siRNA, n = 10, N = 4); CHMP2B-L-mEGFP:ANXA11^{R235Q}-mCh (control siRNA, n = 8, N = 4, ANXA7/ANXA11 siRNA, n = 9, N =4); CHMP2B-L-mEGFP:ANXA11$^{\delta LCD}$-mCh (control siRNA, n = 8, N = 4; ANXA7/ANXA11 siRNA, n = 13, N = 4). Significance calculated via one-way ANOVA with Dunnett's correction, multiple comparisons shown relative to ANXA11-mCh + A7/A11 siRNA.

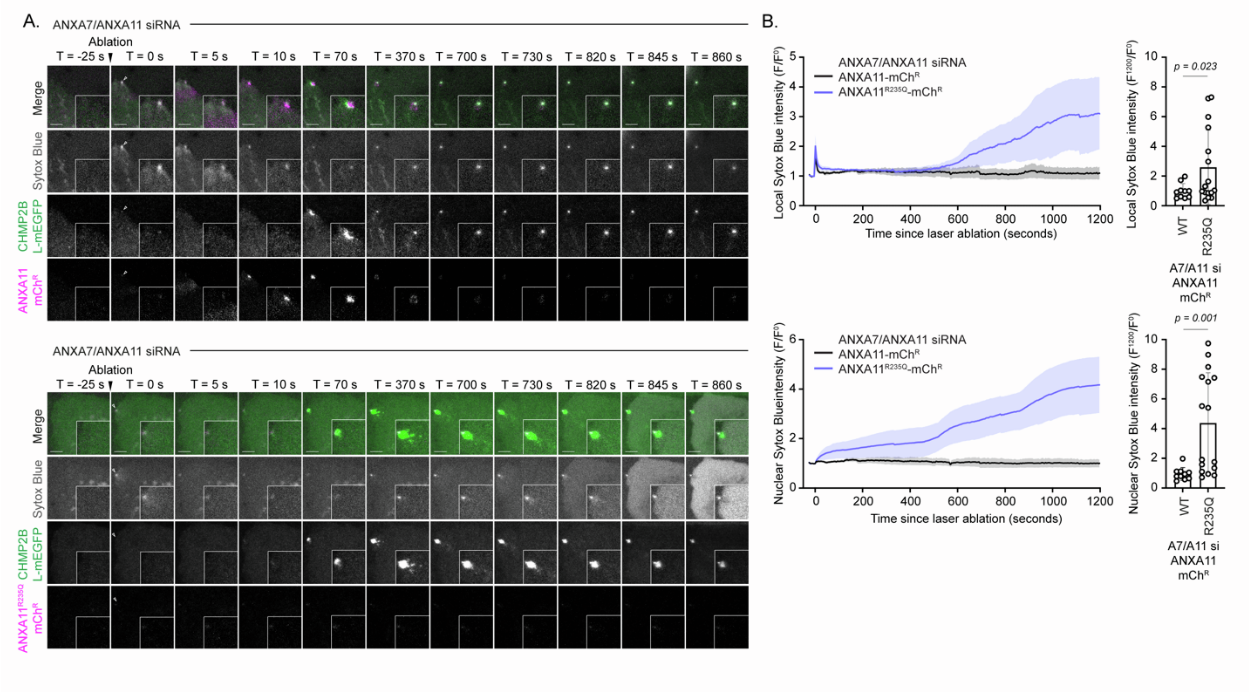

Figure S5. ANXA11^{R235Q}-mCh impairs membrane sealing after damage, related to Figure 4.

(A) Representative image sequences from ANXA7- and ANXA11-siRNA transfected CAL-51 cells stably expressing CHMP2B-L-mEGFP and either ANXA11-mCh^{R} or ANXA11^{R235Q}-mCh^{R}. Cells were incubated in the presence of Sytox Blue and subject to live cell imaging and laser ablation at the plasma membrane. Arrowhead indicates the site of ablation. Scale bar is 5 µm. Note, in the presence of Sytox Blue, laser ablation led to photoconversion of the ibiTreat substrate in the blue and green channels at the site of ablation. This spot did not move with the cells and remained cell distal to the position of the ANXA11-mCh plug. As such, this spot was excluded from regions of quantification.

(B) Quantification of Sytox Blue influx from cells in A, either locally at the site of damage, or in the nucleus. Fluorescence intensities were background corrected and relative to the average of the pre-ablation frames in each case. Traces depict mean ± S.E.M. ANXA11-mCh^{R} (n = 10, N = 4); ANXA11^{R235Q}-mCh^{R} (n = 16, N = 4). The bar graphs depict the local or nuclear PI intensity at 20 minutes after ablation and display mean ± S.D. of all data points. Significance across individual values is presented and was calculated by 2-tailed T-test. See corresponding Mendeley Data Videos 26 and 27.

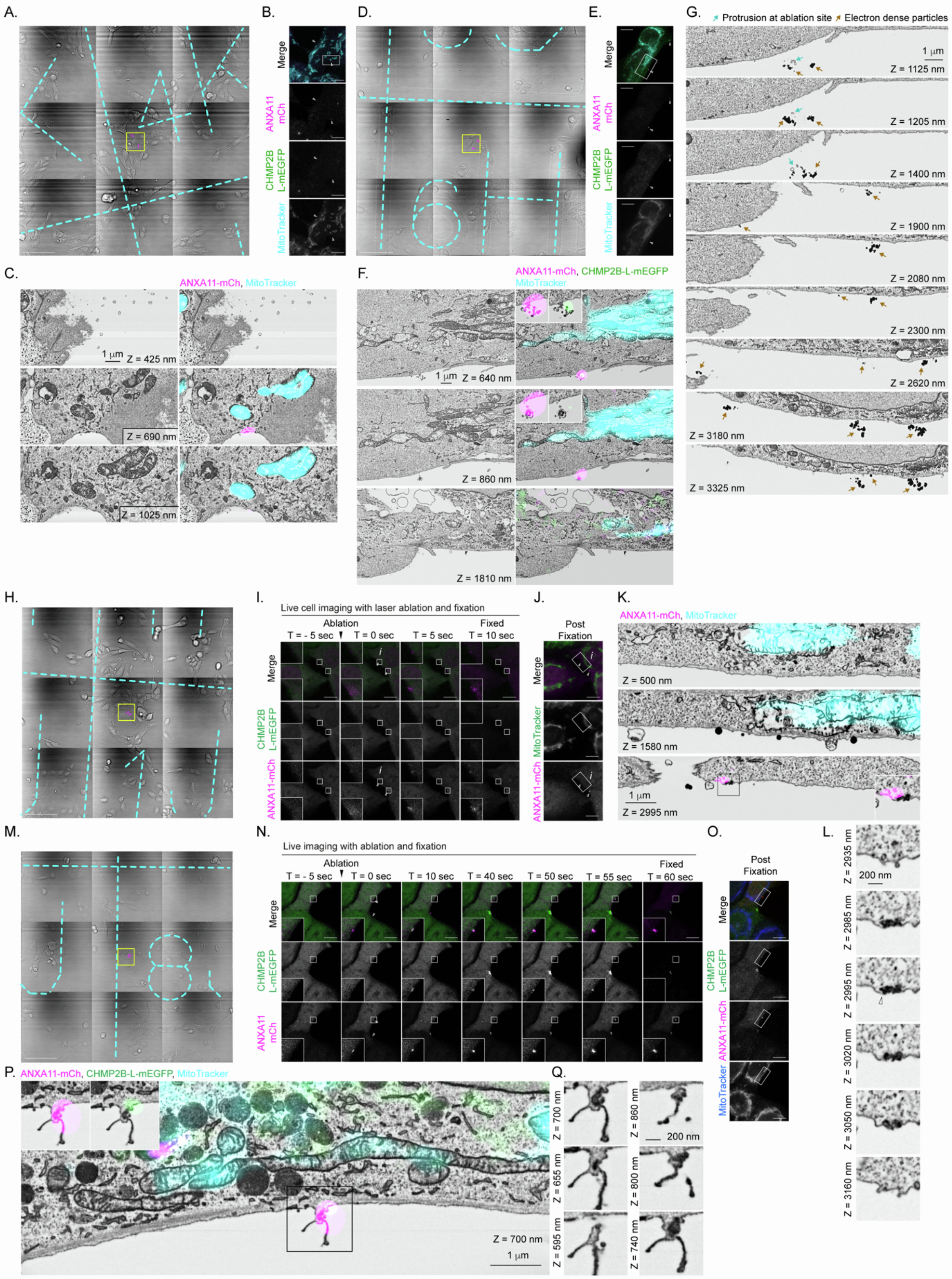

A.
B.
Merge
ANXA11 mCh
CHMP2B L-mEGFP
MitoTracker
C.
ANXA11-mCh, MitoTracker
1 μm
Z = 425 nm
Z = 690 nm
Z = 1025 nm
D.
E.
F.
ANXA11-mCh, CHMP2B-L-mEGFP
MitoTracker
Z = 640 nm
Z = 860 nm
Z = 1810 nm
G.
Protrusion at ablation site
Electron dense particles
Z = 1125 nm
Z = 1205 nm
Z = 1400 nm
Z = 1900 nm
Z = 2080 nm
Z = 2300 nm
Z = 2620 nm
Z = 3180 nm
Z = 3325 nm
H.
I.
Live cell imaging with laser ablation and fixation
Ablation
Fixed
T = - 5 sec
T = 0 sec
T = 5 sec
T = 10 sec
ANXA11-mCh
J.
Post Fixation
K.
Z = 500 nm
Z = 1580 nm
Z = 2995 nm
M.
N.
Live imaging with ablation and fixation
T = 10 sec
T = 40 sec
T = 50 sec
T = 55 sec
T = 60 sec
O.
L.
Z = 2935 nm
200 nm
Z = 2985 nm
Z = 2995 nm
Z = 3020 nm
Z = 3050 nm
Z = 3160 nm
P.
Z = 700 nm
Q.
Z = 655 nm
Z = 595 nm
Z = 860 nm
Z = 800 nm
Z = 740 nm

Figure S6. Volumetric CLEM at sites of laser ablation, related to Figure 5.

(A) Transmitted light imaging of the site of ablation in Figure 5A. Sites of ablation marked (magenta arrows) and the field of view of the site of ablation presented in the timelapse imaging demarcated (yellow). Etched gridlines for re-finding in EM are overlayed (cyan). Scale bar is 100 μm.

(B) A post-fixation light microscopy (LM) Z-stack at the site of ablation (0.12 μm Z-spacing) was acquired to enable registration of LM and EM datasets; Z-position at the ablation point shown. Please note, although ablation was multiplexed (arrowheads), only a single site (boxed) was FIB-milled. Scale bar is 10 μm.

(C) Z-sections from the FIB-SEM datasets in Figure 5A with or without overlay of the MitoTracker and ANXA11-mCh signals after registration of FIB-SEM and LM datasets. The MitoTracker signal was used to align FM and EM volumes and reveal placement of the ANXA11-mCh and/or CHMP2B-L-mEGFP signals in volumetric EM space. Scale bar applies across all panels.

(D) Transmitted light imaging of the site of ablation in Figure 5E. Sites of ablation marked (magenta arrows) and the field of view of the site of ablation presented in the timelapse imaging demarcated (yellow). Etched gridlines for re-finding in EM are overlayed (cyan). Scale bar is 100 μm.

(E) A post-fixation LM Z-stack at the site of ablation (0.12 μm Z-spacing) was acquired to enable registration of LM and EM datasets; Z-position at the ablation point shown. Please note, although ablation was multiplexed (arrowheads), only a single site (boxed) was FIB-milled. Scale bar is 10 μm.

(F) Z-sections from the FIB-SEM datasets in Figure 5E with or without overlay of the MitoTracker, CHMP2B-L-mEGFP and ANXA11-mCh signals. The MitoTracker signal was used to align FM and EM volumes and reveal placement of the ANXA11-mCh and/or CHMP2B-L-mEGFP signals in volumetric EM space, as in (C). Scale bar applies across all panels.

(G) In the reconstruction of the FIB-SEM dataset in Figure 5E – 5H, clusters of highly-electron dense particles were observed at several positions across the surface of the cell. Whilst a cluster was adjacent to the site of ablation, clusters were also found in ablation-distal regions all over the cell's surface. We believe them to be related to the heavy-metal EM staining. Examples provided. These heavy metal clusters were not reconstructed. Scale bar applies to all images in the panel.

(H) Transmitted light imaging of the site of ablation in S6I. Sites of ablation marked (magenta arrows) and the field of view of the site of ablation presented in the timelapse imaging demarcated (yellow). Etched gridlines for re-finding in EM are overlayed (cyan). Scale bar is 100 μm.

(I) Live cell imaging of CAL-51 cells stably expressing ANXA11-mCh and CHMP2B-L-mEGFP, subject to laser ablation at the indicated positions (arrowheads) and fixed after 5 seconds. ANXA11-mCh, but not yet CHMP2B-L-mEGFP recruitment was observed at the site of ablation (enlargements). Site *i* was selected for correlative FIB-SEM imaging. Scale bar is 10 μm.

(J) A post-fixation LM Z-stack at the site of ablation in S6H and S6I (0.12 μm Z-spacing) was acquired to enable registration of Light Microscopy (LM) and EM datasets on the MitoTracker signal; Z-position at the ablation point shown. Scale bar is 10 μm.

(K, L) A Z-section from the FIB-SEM dataset relating to S6H – S6J is presented with the co-registered MitoTracker signals, revealing ANXA11-mCh localised to the site of ablation. Z-slices from the FIB-SEM at the site of ANXA11-mCh recruitment showing a surface-exposed electron dense plug in the damaged membrane are presented in L. Please note the gap between the membrane and the plug at Z = 2995 nm (arrowhead). See corresponding Video S6. In K and the insets in L, the scale bar applies to all panels.

(M) Transmitted light imaging of the site of ablation in S6N. Sites of ablation marked (magenta arrows) and the field of view of the site of ablation presented in the timelapse imaging demarcated (yellow). Etched gridlines for re-finding in EM are overlayed (cyan). Scale bar is 100 μm.

(N) Live cell imaging of CAL-51 cells stably expressing ANXA11-mCh and CHMP2B-L-mEGFP, subject to laser ablation at the indicated positions (arrowheads) and fixed after 55 seconds. CHMP2B-L-mEGFP had been recruited and was observed to localize to the base of an ANXA11-mCh-positive projection at this timepoint. Scale bar is 10 μm.

(O) A post-fixation LM Z-stack at the site of ablation in S6M and S6N (0.12 μm Z-spacing) was acquired to enable registration of Light Microscopy (LM) and EM datasets on the MitoTracker signal; Z-position at the ablation point shown. Scale bar is 10 μm.

(P-Q) A Z-section from the FIB-SEM dataset relating to S6M – S6N is presented with the co-registered MitoTracker signals, revealing ANXA11-mCh and CHMP2B-L-mEGFP localised to a protrusion at the site of ablation. Z-slices from FIB-SEM stacks at the site of ANXA11-mCh and CHMP2B-L-mEGFP recruitment showing an electron dense plug in the damaged and protruding membrane are presented in Q. See corresponding Video S6. In the insets in Q, the scale bar applies to all panels.

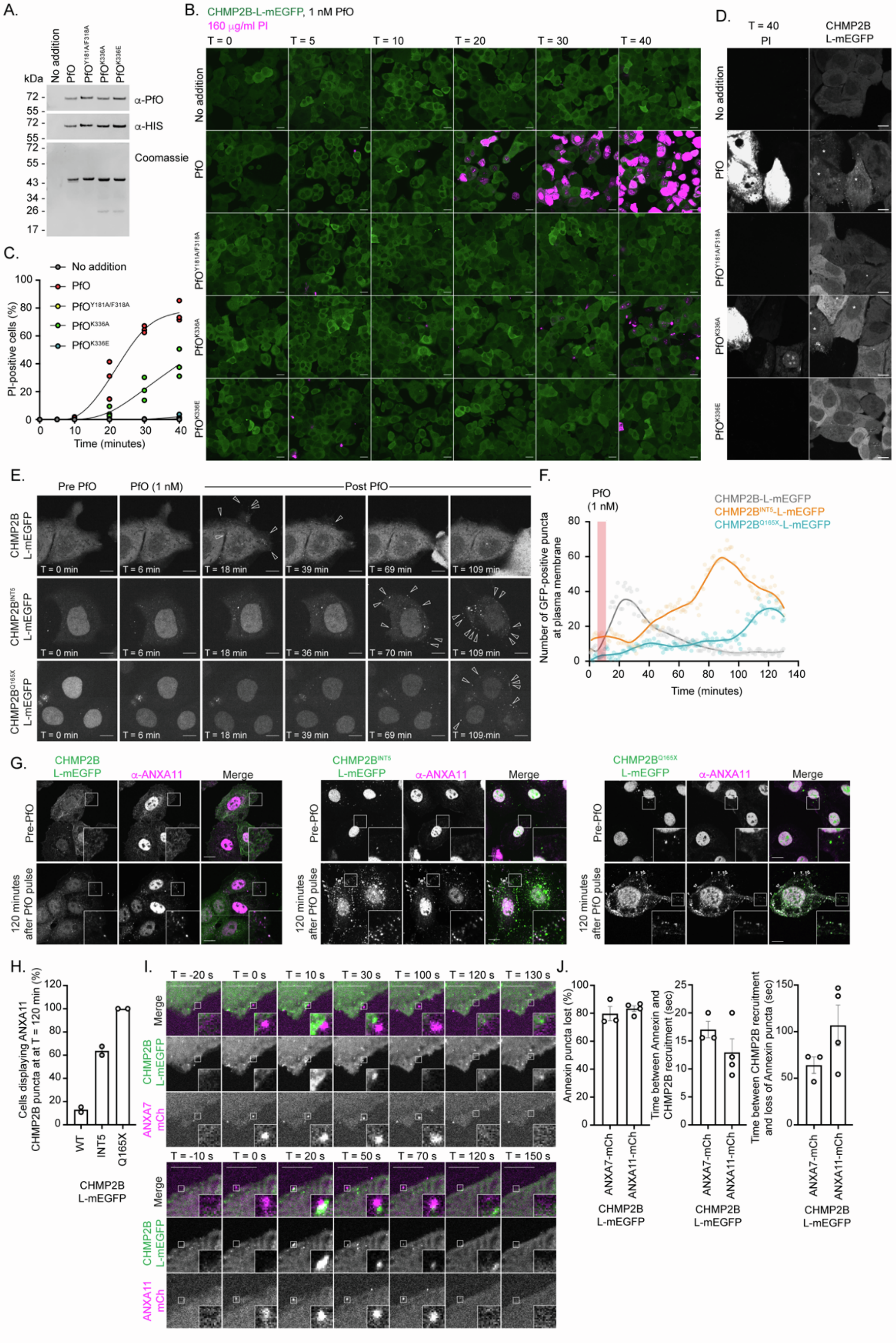
A.
No addition
PfO
PfO Y181A/F318A
PfO K336A
PfO K336E
kDa
α-PfO
α-HIS
Coomassie
B.
CHMP2B-L-mEGFP, 1 nM PfO
160 μg/ml PI
T = 0
T = 5
T = 10
T = 20
T = 30
T = 40
C.
PI-positive cells (%)
Time (minutes)
D.
T = 40
PI
CHMP2B L-mEGFP
E.
Pre PfO
PfO (1 nM)
Post PfO
CHMP2B L-mEGFP
CHMP2B INT5 L-mEGFP
CHMP2B Q165X L-mEGFP
F.
PfO (1 nM)
CHMP2B-L-mEGFP
CHMP2B INT5-L-mEGFP
CHMP2B Q165X-L-mEGFP
Number of GFP-positive puncta at plasma membrane
Time (minutes)
G.
CHMP2B L-mEGFP
α-ANXA11
Merge
Pre-PfO
120 minutes after PfO pulse
H.
Cells displaying ANXA11 CHMP2B puncta at at T = 120 min (%)
WT
INT5
Q165X
CHMP2B L-mEGFP
I.
Merge
CHMP2B L-mEGFP
ANXA7 mCh
ANXA11 mCh
J.
Annexin puncta lost (%)
Time between Annexin and CHMP2B recruitment (sec)
Time between CHMP2B recruitment and loss of Annexin puncta (sec)
ANXA7-mCh
ANXA11-mCh
CHMP2B L-mEGFP

Figure S7. Characterisation of a non-lytic PfO and the response of C-truncated mutants of CHMP2B to PfO-mediated membrane damage, related to Figure 6.

(A) Resolved fractions of recombinant HIS-tagged PfO, $PfO^{Y181A/F316A}$, PfO^{K336A} and PfO^{K336E} were examined by SDS-PAGE and western blotting with antibodies that detect PfO or HIS-Tag or were examined by Coomassie brilliant blue staining.

(B) Representative image sequences of CAL-51 cells stably expressing CHMP2B-L-mEGFP and treated with vehicle or the indicated recombinant PfO proteins at 1 nM in the presence of 160 μg/mL PI and imaged live. T = 0 images were acquired prior to addition of wildtype or mutant PfO. Scale bar is 20 μm.

(C) Quantification of PI entry in cells described in B. Vehicle (n = between 746 and 1335 cells per timepoint; PfO (n = between 725 and 1276 cells per timepoint), $PfO^{Y181A/F316A}$ (n = between 992 and 1373 cells per timepoint; datapoints overlap with the no-addition datapoints), PfO^{K336A} (n = between 931 and 1357 cells per timepoint) and PfO^{K336E} (n = between 1037 and 1382 cells per timepoint) from N = 3 independent experiments. A Lowess curve was used to fit the data.

(D) Examination of CHMP2B-L-mEGFP puncta at the plasma membrane of cells from B. CHMP2B-L-mEGFP puncta at the plasma membrane are present in cells with PI influx (indicated by stars). Scale bar is 10 μm.

(E, F) Live cell imaging (E) and quantification (F) of CHMP2B puncta formation (indicated by arrowheads) at the plasma membrane of CAL-51 cells stably expressing CHMP2B-L-mEGFP, $CHMP2B^{INT5}$-L-mEGFP or $CHMP2B^{Q165X}$-L-mEGFP, imaged for 5 minutes (pre-PfO) treated with 1 nM PfO for 5 minutes and chased into fresh media (post-PfO). Scale bar is 10 μm. A Lowess curve was used to fit the data in F. See corresponding Mendeley Data Videos 28 - 30.

(G, H) CAL-51 cells stably expressing CHMP2B-L-mEGFP, $CHMP2B^{INT5}$-L-mEGFP or $CHMP2B^{Q165X}$-L-mEGFP were treated with 1 nM PfO for 5 minutes, washed and chased into fresh media for 2 hours. Cells fixed and stained with antibodies raised against ANXA11. Cells displaying > 3 puncta at the plasma membrane where endogenous ANXA11 colocalised with the mEGFP was scored (CHMP2B-L-mEGFP, n = 92, N = 2; $CHMP2B^{INT5}$-L-mEGFP, n = 77, N = 2; $CHMP2B^{Q165X}$-L-mEGFP, n = 16, N = 2). In G, scale bar is 10 μm. In H, graph displays mean.

(I) Representative images of CAL-51 cells stably expressing CHMP2B-L-mEGFP and either ANXA7-mCh or ANXA11-mCh that had been treated with a 5-minute pulse of 1 nM PfO, chased into fresh media and imaged live. T = 0 was set at the time of ANXA7-mCh or ANXA11-mCh puncta formation. See corresponding Mendeley Data Videos 31 and 32. Scale bar is 15 μm.

(J) Quantification of recruitment dynamics and release of ANXA7-mCh or ANXA11-mCh puncta in the first 6 minutes of imaging in S7I. For calculation of the percentage of ANXA7-mCh and ANXA11-mCh lost after CHMP2B-L-mEGFP recruitment, we followed n = 61 puncta from 12 cells across N = 3 PfO additions for ANXA7-mch and n = 44 puncta from 13 cells across N = 4 PfO additions for ANXA11-mCh. Graph displays mean ± S.E.M. For calculation of the time between ANXA7-mCh and CHMP2B-L-mEGFP recruitment, or ANXA11-mCh and CHMP2B-L-mEGFP recruitment, we followed n = 56 quantifications across 12 cells from N = 3 PfO additions for ANXA7-mCh and n = 40 events from 13 cells across N = 4 PfO additions for ANXA11-mCh. Graph displays mean ± S.E.M. Note, in some cases, CHMP2B-L-mEGFP was not clearly detectable, reducing the n number from the total number of puncta counted. For calculation of the time between CHMP2B-L-mEGFP recruitment and Annexin puncta lost, we followed n = 42 events from 12 cells across N = 3 PfO additions for ANXA7-mCh and n = 33 events from 13 cells across N = 4 PfO additions for ANXA11-mCh. Graph displays mean ± S.E.M. Note, in some cases, Annexin puncta were not released in the time frame of imaging, reducing the n-number from the total number of puncta counted. No significant differences between ANXA7-mCh and ANXA11-mCh expressing cell lines were observed, as calculated by unpaired two-tailed T-test.
